# The feedback loop between MTA1 and MTA3/TRIM21 modulates stemness of breast cancer in response to estrogen

**DOI:** 10.1038/s41419-024-06942-w

**Published:** 2024-08-17

**Authors:** Jingyao Zhang, Yinuo Wang, Jingjing Zhang, Xin Wang, Jiaxiang Liu, Miaomiao Huo, Ting Hu, Tianyu Ma, Die Zhang, Yu Li, Chang Guo, Yunkai Yang, Min Zhang, Baowen Yuan, Hao Qin, Xu Teng, Tianyang Gao, Xinhui Hao, Hefen Yu, Wei Huang, Binghe Xu, Yan Wang

**Affiliations:** 1 State Key Laboratory of Molecular Oncology, National Cancer Center/National Clinical Research Center for Cancer/Cancer Hospital, Chinese Academy of Medical Sciences and Peking Union Medical College, Beijing, China; 2 Department of Breast Surgical Oncology, National Cancer Center/National Clinical Research Center for Cancer/Cancer Hospital, Chinese Academy of Medical Sciences and Peking Union Medical College, Beijing, China; 3 Beijing Key Laboratory of Cancer Invasion and Metastasis Research, Department of Biochemistry and Molecular Biology, School of Basic Medical Sciences, Capital Medical University, Beijing, China; 4 Key Laboratory of Immune Microenvironment and Disease (Ministry of Education), Department of Biochemistry and Molecular Biology, School of Basic Medical Sciences, Tianjin Medical University, Tianjin, China; 5 Department of Medical Oncology, National Cancer Center/National Clinical Research Center for Cancer/Cancer Hospital, Chinese Academy of Medical Sciences and Peking Union Medical College, Beijing, China

**Keywords:** Cancer stem cells, Mesenchymal migration

## Abstract

The metastasis-associated protein (MTA) family plays a crucial role in the development of breast cancer, a common malignancy with a high incidence rate among women. However, the mechanism by which each member of the MTA family contributes to breast cancer progression is poorly understood. In this study, we aimed to investigate the roles of MTA1, MTA3, and tripartite motif-containing 21 (TRIM21) in the proliferation, invasion, epithelial-mesenchymal transition (EMT), and stem cell-like properties of breast cancer cells in vivo and in vitro. The molecular mechanisms of the feedback loop between MTA1 and MTA3/TRIM21 regulated by estrogen were explored using Chromatin immunoprecipitation (ChIP), luciferase reporter, immunoprecipitation (IP), and ubiquitination assays. These findings demonstrated that MTA1 acts as a driver to promote the progression of breast cancer by repressing the transcription of tumor suppressor genes, including TRIM21 and MTA3. Conversely, MTA3 inhibited MTA1 transcription and TRIM21 regulated MTA1 protein stability in breast cancer. Estrogen disrupted the balance between MTA1 and MTA3, as well as between MTA1 and TRIM21, thereby affecting stemness and the EMT processes in breast cancer. These findings suggest that MTA1 plays a vital role in stem cell fate and the hierarchical regulatory network of EMT through negative feedback loops with MTA3 or TRIM21 in response to estrogen, supporting MTA1, MTA3, and TRIM21 as potential prognostic biomarkers and MTA1 as a treatment target for future breast cancer therapies.

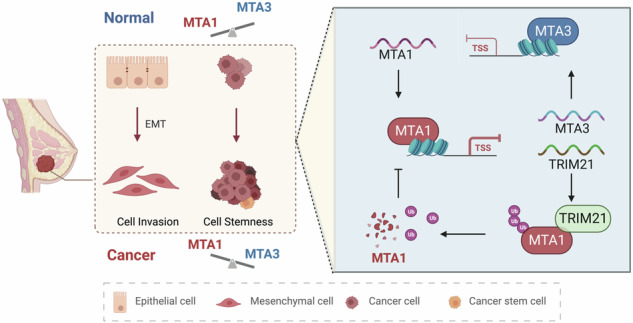

## Introduction

Breast cancer seriously affects the health of women worldwide, which accounts for 31% of female malignancies and 15% of female deaths from malignancies [1]. Tumor metastasis is an imperative reason of poor survival in breast cancer. Cancer stemness and epithelial-mesenchymal transition (EMT) in malignant tumors are important factors influencing tumor metastasis [2]. However, the exact molecular mechanisms underlying tumor metastasis remain poorly understood.

EMT, a multistep dynamic process, plays a crucial role in the progression of malignant tumors. The transcription factors regulating EMT, including snail family transcriptional repressor 1 (SNAI1), twist family bhlh transcription factor 1 (Twist1), zinc finger e-box binding homeobox 1 (ZEB1) and ZEB2, regulate cancer migration, invasion, and metastasis [3]. EMT drives the stemness of cancer cells, and there is a pronounced overlap in the transcriptomic profiles of cells that undergo EMT and cancer stem cells [4–6]. Cancer cells acquire stemness by promoting EMT to allow invasive metastasis through epigenetic modulation [7]. Previous studies have reported that epithelial cell adhesion molecule (EpCAM) promotes cancer cell self-renewal and bone metastasis [8, 9].

Nucleosome remodeling deacetylase complex (NuRD) and histone deacetylase (HDAC) act as chromatin remodeling complexes that repress transcription by regulating histone deacetylation [10]. The MTA family, including MTA1, MTA2, and MTA3, regulates the transcription of downstream genes that are essential components of the NuRD complex [11]. The N-terminal regions of MTA1, MTA2, and MTA3 are highly homologous, whereas the C-terminal contains a unique proline-rich region only in MTA1. Differences in the C-terminus are responsible for the different roles of MTA1 and MTA3 in tumors [12, 13]. Unlike MTA1 and MTA3, little change has been reported in MTA2 expression during tumorigenesis [14]. Recent studies have revealed that MTA1 is overexpressed in various tumors, including breast, prostate, cervical, and liver cancers [15–18]. MTA1 is involved in various steps of the malignant transformation, containing invasion, EMT, DNA damage, angiogenesis, inflammation, metastasis, and drug resistance. It exerts transcriptional repression by binding to the NuRD complex and blocks estrogen receptor 1 (ESR1) transcription stimulated by estrogen [19].

MTA3 exerts transcriptional repression mainly through the NuRD complex to suppress cancer cell stemness and metastasis and ultimately inhibit tumorigenesis [16, 20]. Our previous study demonstrated that the MTA3/GATA binding protein 3 (GATA3)/G9A complex can transcriptionally repress the transcription factor zinc finger e-box binding homeobox 2 (ZEB2) to suppress breast carcinogenesis [16]. However, the mechanisms by which MTA1 and MTA3 play important roles in stem cell-like properties and EMT in breast cancer remain poorly understood.

In this study, we investigated whether MTA1 is overexpressed in breast cancer tissues and whether it correlates with poor survival in patients with breast cancer. MTA3 expression is progressively downregulated in breast cancer, and the absence of MTA3 promotes disease progression in breast cancer. We found that MTA1 and MTA3 are recognized by specific transcription factors and play opposing roles in breast cancer.

## Results

### Upregulation of MTA1 expression or downregulation of MTA3 expression correlates with breast cancer progression

**Fig. 1:**
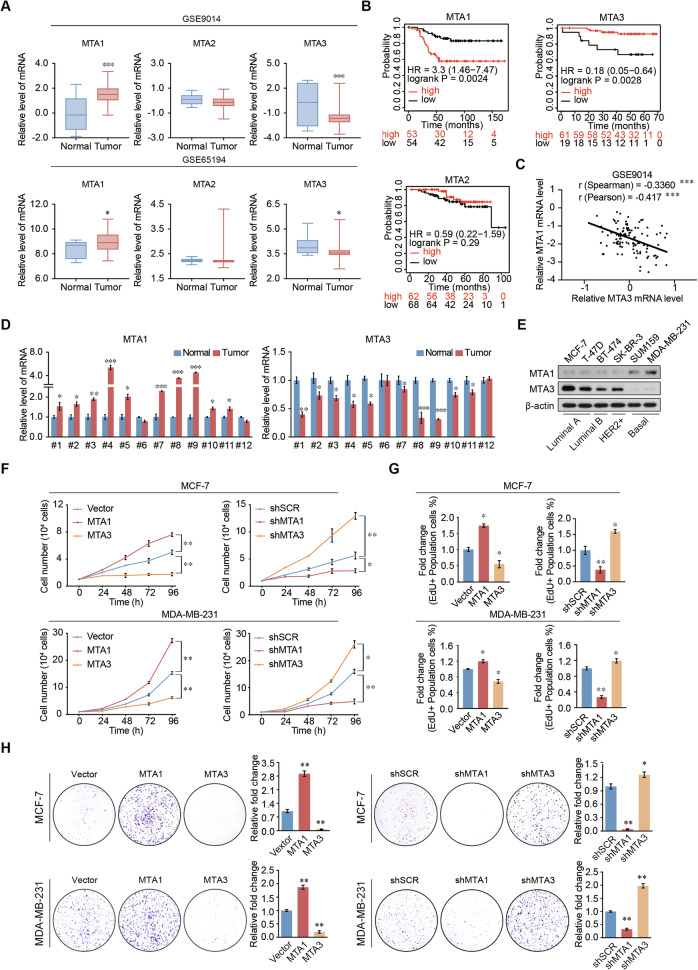
MTA1 is upregulated in breast cancer and promotes the proliferation of breast cancer cells. **A** Analysis of MTA family expression using the GEO dataset indicated that MTA1 was increased and MTA3 was decreased in breast cancer samples compared to normal tissues. **B** Survival analysis demonstrated that high expression of MTA1 indicates a good prognosis, and high expression of MTA3 indicates a poor prognosis. **C** MTA1 expression was negatively correlated with MTA3 expression using the analysis of public datasets. **D** The expression of MTA1 and MTA3 was analyzed by qRT-PCR using total RNA extracted from paired samples of 12 breast cancer tissues and adjacent normal breast tissues. **E** Lysates of different human breast cancer cell lines were subjected to western blotting to examine MTA1 and MTA3 protein expression levels. **F** Growth curve analysis of MCF-7 and MDA-MB-231 cells after overexpression or knockdown of MTA1 or MTA3. **G** EdU assays in MCF-7 and MDA-MB-231 cells transfected with vector, MTA1, MTA3, shSCR, shMTA1, or shMTA3. **H** Colony formation experiments after transfection with vector, MTA1, MTA3, shSCR, shMTA1, and shMTA3 in MCF-7 and MDA-MB-231. Error bars represent the minimum to maximum in **A** and the mean ± SD in **D**, **F**–**H**. *p < 0.05, **p < 0.01, ***p < 0.001; two-tailed unpaired *t*-test.

Gene Expression Omnibus (GEO) datasets analysis indicated that MTA1 expression was upregulated in breast cancer, MTA3 expression was decreased in breast cancer (Fig. 1A and Supplementary Fig. 1A). Patients with high MTA1 expression-breast cancer had poor prognosis. In contrast, patients with high MTA3 expression showed longer-term survival (Fig. 1B). MTA1 expression was significantly negatively correlated and MTA3 expression in breast cancer (Fig. 1C and Supplementary Fig. 1B). The results demonstrated that MTA1 expression was significantly increased in breast cancer tissues, while MTA3 expression was higher in adjacent tissues (Fig. 1D).

The protein expression level of MTA1 was low in MCF-7 cells and high in MDA-MB-231 cells. Conversely, the protein expression level of MTA3 was high in MCF-7 cells and low in MDA-MB-231 cells (Fig. 1E). MCF-7 and MDA-MB-231 cells were transfected with corresponding lentiviruses to generate MTA1 overexpression or knockdown cells and MTA3 overexpression or knockdown cells (Supplementary Fig. 1C). Growth curve analysis showed that MTA1 significantly promoted breast cancer cell growth, whereas MTA3 inhibited cell growth (Fig. 1F). The 5-ethynyl-2′-deoxyuridine (EdU) results showed that MTA1 knockdown or MTA3 overexpression caused a significant decrease in EdU-labeled breast cancer cells, and MTA1 overexpression or MTA3 knockdown caused the opposite effect (Fig. 1G and Supplementary Fig. 1D, E). MTA1 overexpression or MTA3 knockdown increased colony numbers with the comparison of vector group or shScramble (shSCR), whereas MTA1 knockdown or MTA3 gain-of-function decreased this effect in breast cancer cells (Fig. 1H). These results suggest that MTA1 stimulates the proliferation of breast cancer cells, whereas MTA3 exerts an opposite effect.

### MTA1 and MTA3 regulate breast cancer cell invasion and stemness in opposite ways

**Fig. 2:**
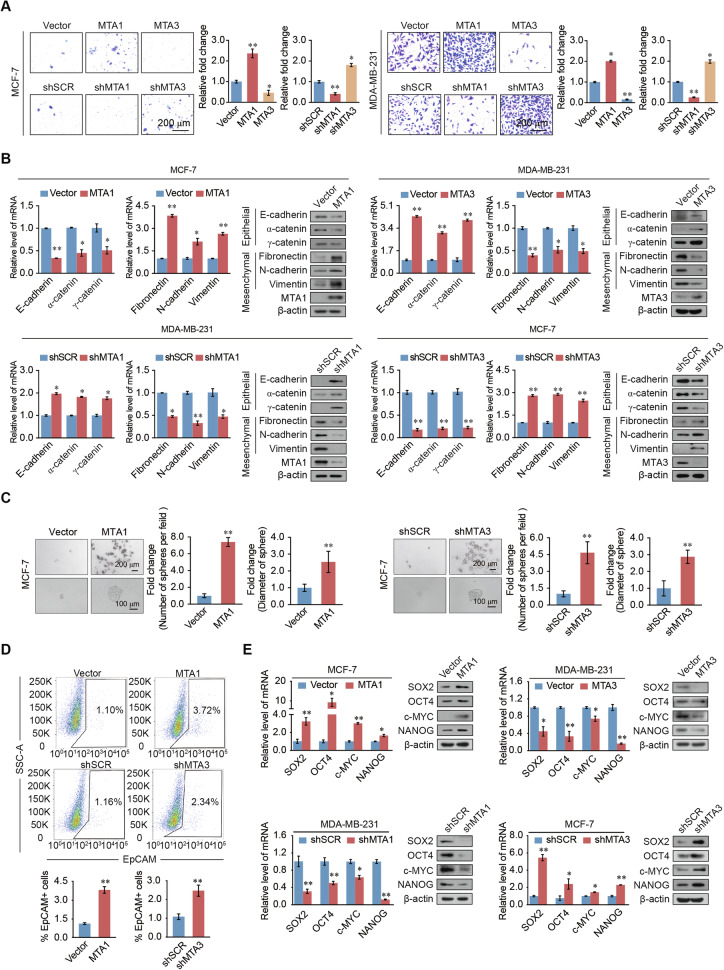
MTA1 promotes migration, invasion, EMT, and stemness in breast cancer cells, whereas MTA3 exerts the opposite effects. **A** Transwell invasion assays performed in MCF-7 and MDA-MB-231 cells after overexpression or knockdown of MTA1 or MTA3, respectively. **B** mRNA and protein expression levels of epithelial and mesenchymal markers, as determined by RT-qPCR and western blotting, respectively, after overexpression of MTA1 or knockdown of MTA3 in MCF-7 cells, knockdown of MTA1, or overexpression of MTA3 in MDA-MB-231 cells. **C** Representative images of spheroid formation in suspension culture after 15 days of MTA1 overexpression or MTA3 knockdown in MCF-7 cells. The number of spheres per field and the diameters of the spheres were statistically analyzed. **D** Flow cytometry results of EpCAM-stained MDA-MB-231 cells stably overexpressing of MTA1 or knockdown of MTA3. **E** RT-qPCR and western blotting to determine the mRNA and protein expression levels of stem cell markers (SOX2, OCT-4, c-MYC, and NANOG) after knockdown or overexpression of MTA1 or MTA3. Error bars represent the mean ± SD in **A**–**E**. *p < 0.05, **p < 0.01; two-tailed unpaired *t*-test.

To verify the tumorigenic function of MTA1 and MTA3 in breast cancer cells, Transwell and wound healing assays were carried out. MTA1 overexpression or MTA3 knockdown promoted breast cancer invasion, whereas knockdown of MTA1 or MTA3 overexpression had the opposite effect (Fig. 2A). Migration assays revealed that MTA1 overexpression or MTA3 knockdown resulted into an increase in the migration of breast cancer cells, whereas MTA1 knockdown or MTA3 overexpression induced the opposite effect (Supplementary Fig. 2A). Real-time quantitative PCR (RT-qPCR) and western blotting showed that MTA1 overexpression or MTA3 knockdown downregulated the expression of epithelial markers, containing E-cadherin, α- and γ-catenin, and upregulated the expression of mesenchymal markers fibronectin, N-cadherin, and vimentin. MTA1 knockdown and MTA3 overexpression had opposite effects on the expression of EMT markers (Fig. 2B). These findings confirm that MTA1 is essential for controlling EMT and promoting breast cell invasion.

Overexpression of MTA1 or MTA3 knockdown led to a significant increase of sphere-forming efficiency and sphere diameter compared to the control group, whereas MTA1 knockdown or overexpression of MTA3 reduced these levels (Fig. 2C and Supplementary Fig. 2B). Flow cytometry assays identified that EpCAM-positive cells increased after MTA1 overexpression or MTA3 knockdown (Fig. 2D). RT-qPCR and western blotting identified that the expression of stem cell markers sry-box transcription factor 2 (SOX2), pou class 5 homeobox 1 (OCT4), myc proto-oncogene, bhlh transcription factor (c-MYC), and nanog homeobox (NANOG) were upregulated by MTA1 overexpression or MTA3 knockdown in breast cancer cells. MTA1 knockdown and MTA3 overexpression had the opposite effects (Fig. 2E). These results determine that MTA1 promotes breast cancer cell migration, invasion, and stemness in vitro and that the role of MTA3 in these phenotypes was the opposite of that of MTA1.

### Genome-wide transcription targets for MTA1 and MTA3

**Fig. 3:**
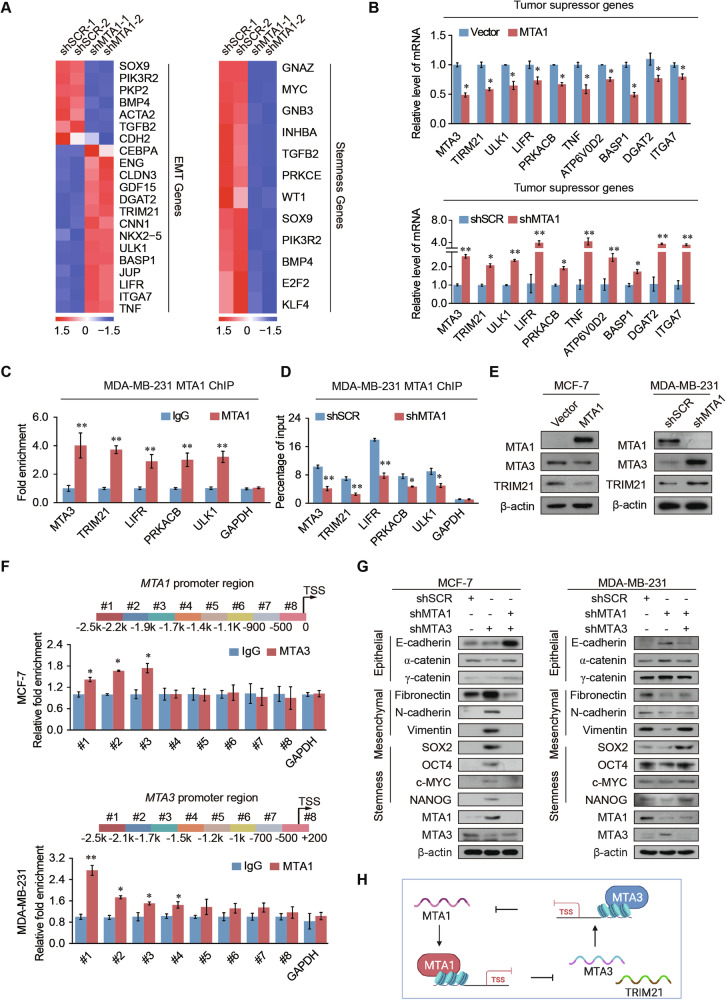
MTA1 regulates tumor suppressor, EMT, and stemness pathways. **A** Heatmap of known EMT-associated-and tumor stemness-associated genes from differential gene analysis of shMTA1 and shSCR cells RNA sequencing. **B** RT-qPCR analysis of selected tumor suppressor genes (*MTA3, TRIM21*, *ULK1*, *LIFR*, *PRKACB*, *TNF*, *ATP6V0D2*, *BASP1*, *DGAT2*, and *ITGA7*) after overexpression of MTA1 in MCF-7 cells or knockdown of MTA1 in MDA-MB-231 cells. mRNA expression levels of the target genes were normalized to that of *GAPDH* levels. **C** qChIP analysis of indicated genes in MDA-MB-231 cells. GAPDH was used as internal control. **D** MDA-MB-231 cells were infected with shSCR and shMTA1 lentiviruses. qChIP analysis of tumor transcription factor recruitment was performed using an MTA1 antibody, with GAPDH as an internal control. **E** Western blotting demonstrated that overexpression MTA1 downregulated MTA3 and TRIM21 protein expression levels in MCF-7 cells. The protein expression levels of MTA3 and TRIM21 increase in MDA-MB-231 cells after stably transfected with shMTA1. **F** Primer pairs #1–8 were synthesized to cover the promoter region of *MTA1*, and a qChIP-based promoter walking experiment was performed using MCF-7 cells (up). Primer pairs #1–8 were synthesized to cover the promoter region of *MTA3*, and a qChIP-based promoter walking experiment was performed using MDA-MB-231 cells (bottom). **G** Western blotting results for epithelial, mesenchymal, and stemness markers after the simultaneous knockdown of MTA1 and MTA3 in MDA-MB-231 and MCF-7 cells. **H** Schematic overview of the transcriptionally regulatory between MTA1 and MTA3. Error bars represent the mean ± SD in **B**–**D**, **F**. *p < 0.05, **p < 0.01; two-tailed unpaired *t*-test.

We conducted RNA-seq on MCF-7 cells with MTA1 knockdown to investigate its role in regulating breast cancer malignancy. Our analysis identified 1117 upregulated and 341 downregulated differentially expressed genes (DEGs) in the cancer cells compared to control cells (Supplementary Fig. 3A). Kyoto Encyclopedia of Genes and Genomes (KEGG) pathway analysis of DEGs showed that the upregulated DEGs were mainly enriched in the cytokine receptor interaction, Toll-like receptor, nuclear factor kappa B (NF-κB), and tumor necrosis factor (TNF) pathways, which are important for tumorigenesis and progression. The downregulated DEGs were mainly related to the cell cycle, cyclic adenosine 3’,5’-monophosphate (cAMP), AMP-activated protein kinase (AMPK), and especially the pathway of EMT and focal adhesion pathways, which are related to cancer metastasis (Supplementary Fig. 3B, C). The DEGs were strongly enriched in EMT- and stem cell-related genes (Fig. 3A) and included multiple tumor suppressors. The mRNA expression levels of tumor suppressors *MTA3, TRIM21*, unc-51 like autophagy activating kinase 1 (*ULK1*), lif receptor subunit alpha (*LIFR*), protein kinase cAMP-activated catalytic subunit beta (*PRKACB*), *TNF*, ATPase H+ transporting V0 subunit D2 (*ATP6V0D2*), brain abundant membrane attached signal protein 1 (*BASP1*), diacylglycerol o-acyltransferase 2 (*DGAT2*), and integrin subunit alpha 7 (*ITGA7*) were upregulated in MTA1 knockdown cells, and the mRNA expression levels of these genes were in a decrease after overexpression MTA1 (Fig. 3B). Chromatin immunoprecipitation (ChIP) assay was used to identify that MTA1 was enriched in the promoters of the *MTA3*, *TRIM21*, *LIFR*, *PRKACB* and *ULK1* to inhibit the transcription of these genes (Fig. 3C). The recruitment of MTA1 to these tumor suppressors was attenuated when MTA1 was knocked down (Fig. 3D). The protein expression levels of MTA3 and TRIM21 increased after MTA1 knockdown, whereas they decreased after MTA1 overexpression (Fig. 3E). Quantitative ChIP (qChIP)-based promoter walk results showed significant enrichment of MTA3 in three promoter upstream from −2500 to −1700 in the promoter of *MTA1*. MTA1 was enriched in the *MTA3* promoter upstream from −2500 to −1200 bp (Fig. 3F). These results recommend that MTA1 hinders the expression of tumor suppressor genes, including *MTA3* and *TRIM21*, and influences the EMT and stem cells.

After MTA3 knockdown, 524 DEGs were upregulated and 245 DEGs were downregulated (Supplementary Fig. 3D). The DEGs were significantly enriched in pathways such as oxidative phosphorylation, the estrogen signaling pathway, aging, and metabolism (Supplementary Fig. 3E). Gene set enrichment analysis (GSEA) showed that the DEGs were enriched in EMT and focal adhesion pathways (Supplementary Fig. 3F). MTA1 expression decreased after MTA3 knockdown but increased after MTA3 overexpression (Supplementary Fig. 1C). Together, these results suggest that MTA1 promotes EMT and stemness in breast cancer, while MTA3 exerts an opposite effect.

Knockdown of MTA3 upregulated the expression of mesenchymal and stemness markers and repressed the expression of epithelial markers. These effects were restored by the simultaneous MTA1 knockdown in breast cancer cells. Knockdown of MTA1 improved the expression levels of epithelial markers and declined the expression levels of mesenchymal and stemness markers, whereas concurrent knockdown of MTA1 and MTA3 rescued the expression levels of these markers (Fig. 3G). These findings suggest a mutual transcriptional regulation between MTA1 and MTA3, indicating a negative feedback crosstalk mechanism (Fig. 3H).

### MTA1 promotes breast cancer stemness in vivo

**Fig. 4:**
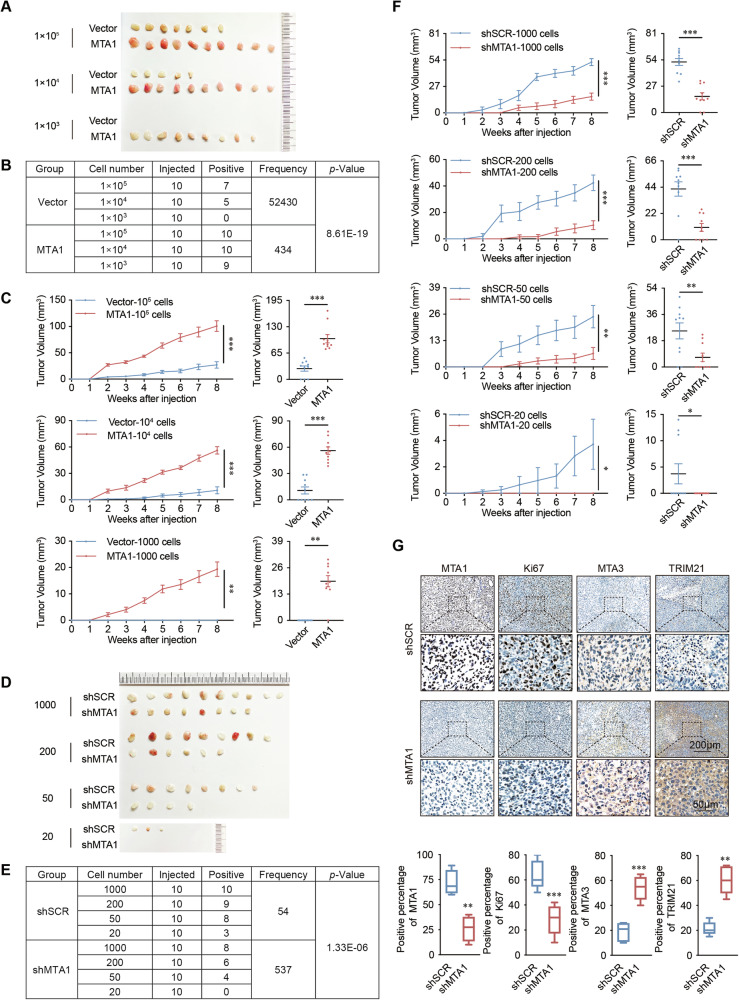
MTA1 promotes the growth of breast cancer xenografts in NOD/SCID mice by promoting the development of cancer stemness. **A** The limited-dilution tumorigenic assay in mice showed that MTA1 promotes breast cancer stemness. Stable overexpression of MTA1 and the control group were constructed in MCF-7 cells. MTA1-overexpressing and vector cells were injected into the fourth pair of mammary fat pads of NOD/SCID female mice (*n* = 10 per group). **B** ELDA analysis indicates that the frequency of cancer stem cells increased after overexpression of MTA1. **C** Tumor growth curves of different numbers (10^5^, 10^4^, 1000) of MTA1-overexpressing and vector cells over the indicated time periods. Endpoint tumor volume of mice with different cell numbers in the overexpression MTA1 group and vector groups. **D** MTA1 knockdown suppressed the stemness of breast cancer in vivo. MDA-MB-231 cells stably transfected with shSCR and shMTA1 were injected into the fourth pair of mammary fat pads of NOD/SCID female mice (*n* = 10 per group). **E** ELDA analysis indicated that MTA1 knockdown reduced the frequency of cancer stem cells at different dilutions (1 000, 200, 50, and 20) of MDA-MB-231-shSCR and MDA-MB-231-shMTA1 cells (*n* = 10 per group). **F** Tumor growth curves of NOD/SCID mice injected with MDA-MB-231-shSCR or MDA-MB-231-shMTA1 tumors with different cell numbers. Tumor volume with different numbers of MDA-MB-231-shSCR or MDA-MB-231-shMTA1 cells at the endpoint. **G** MTA1, Ki67, MTA3, and TRIM21 staining was performed by immunohistochemistry on sections of shSCR and shMTA1 mice from tumor initiation in vitro experiments. Error bars represent the mean ± SEM in **C** and **F**, and min to max in **G**. *p < 0.05, **p < 0.01, ***p < 0.001; two-tailed unpaired *t*-test.

The results of limited-dilution tumorigenic assays in NOD/SCID mice indicated that the overexpression of MTA1 significantly improved tumor initiation. The frequency of breast cancer stem cells was obviously higher in the MTA1 overexpression group with the comparison of control group (Fig. 4A, B). The tumor growth speed of mice in MTA1 overexpression group was significantly faster and the volume and weight of tumor in this group were significantly larger and heavier with the comparison of control group (Fig. 4C and Supplementary Fig. 4A). MTA1 knockdown decreased the frequency of tumor-initiating cells (Fig. 4D, E) and significantly inhibited the growth of breast cancer (Fig. 4F and Supplementary Fig. 4B).

Mice tumors were collected for immunohistochemical staining to detect the protein expression levels of MTA1, MTA3, TRIM21, and Ki67. The protein expression levels of MTA3 and TRIM21 were significantly increased in the tumors of mice in the shRNA-MTA1 group compared to the control group (Fig. 4G). These results suggest that MTA1 plays a crucial role in inducing stemness in breast cancer and promoting tumorigenesis in vivo.

### TRIM21 inhibits proliferation, invasiveness, EMT, and stem-like properties of breast cancer

**Fig. 5:**
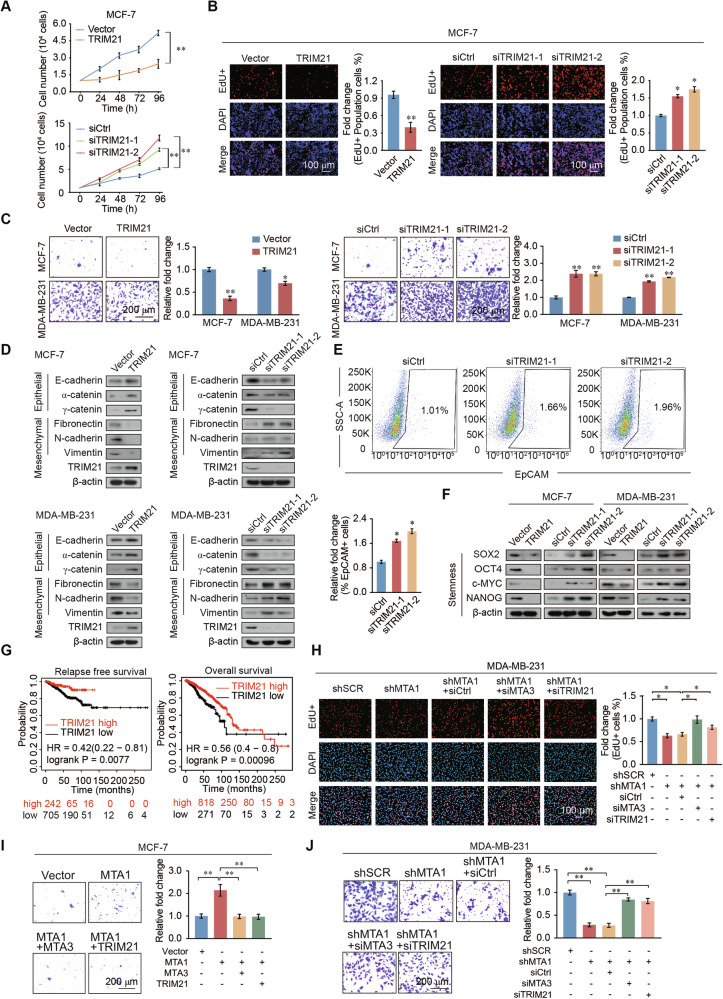
TRIM21 suppresses proliferation, invasion, EMT and stemness of breast cancer cells. **A** Cell counting assays indicated that TRIM21 inhibits breast cancer cell growth in MCF-7 cells. Knockdown of TRIM21 actives breast cancer cell growth. **B** EdU assays performed on MCF-7 and MDA-MB-231 cells transfected with TRIM21, siCtrl, siTRIM21-1, or siTRIM21-2. siCtrl, siControl. **C** Transwell assays indicated that TRIM21 inhibits invasion in MCF-7 and MDA-MB-231 cells with overexpression it and actives invasion in TRIM21 knockdown cells. siCtrl, siControl. **D** Western blot results for the expression of epithelial and mesenchymal markers after overexpression or knockdown of TRIM21 in MCF-7 and MDA-MB-231 cells. **E** Percentage of EpCAM-positive cells assessed by flow cytometry were increased after TRIM21 knockdown in MDA-MB-231 cells. **F** Protein expression levels of the stemness markers SOX2, OCT4, NANOG, and c-MYC in MCF-7 and MDA-MB-231 cells overexpression or knockdown of TRIM21. **G** RFS and OS analyses demonstrated that breast cancer patients with high TRIM21 expression had a better prognosis than those with low expression in. **H** Proliferative ability of MDA-MB-231 cells transfected with siRNA and shRNA viruses determined using the EdU assays. **I** The invasive phenotype was detected in MCF-7 cells transfected with the Vector, MTA1, MTA3, and TRIM21. **J** Transwell assays to identify the invasiveness of MDA-MB-231 cells transfected with siRNA and shRNA viruses. Error bars represent the mean ± SD in **A**–**C**, **E**, **H–J**. *p < 0.05, **p < 0.01; two-tailed unpaired *t*-test.

To explore the role of TRIM21 in breast cancer, we performed cell count assays, which revealed that increased breast cancer cell proliferation after TRIM21 knockdown, whereas overexpression of TRIM21 decreased their proliferation (Fig. 5A and Supplementary Fig. 5A). Furthermore, EdU assays revealed that TRIM21 overexpression significantly decreased the percentage of EdU-positive cells, whereas TRIM21 knockdown increased the proportion compared with that observed in control cells (Fig. 5B and Supplementary Fig. 5B). TRIM21 overexpression inhibited invasiveness, whereas knockdown of TRIM21 promoted invasiveness in breast cancer cells (Fig. 5C). Western blotting revealed that TRIM21 overexpression upregulated the expression levels of epithelial markers and downregulated the expression levels of mesenchymal markers. TRIM21 knockdown reduced the expression of epithelial markers and upregulated that of mesenchymal markers (Fig. 5D). Flow cytometry assays showed that after TRIM21 knockdown, the proportion of EpCAM-positive cells increased significantly (Fig. 5E). Overexpression of TRIM21 resulted in a significant reduction in the protein expression levels of the stemness markers SOX2, OCT4, c-MYC, and NANOG, whereas TRIM21 knockdown had the opposite effect (Fig. 5F). Breast cancer patients with high TRIM21 expression have favorable relapse free survival (RFS) and overall survival (OS) compared with those with low TRIM21 expression (Fig. 5G). The same results apply to rectum adenocarcinoma, stomach adenocarcinoma, and endometrial cancer (Supplementary Fig. 5C, D). These results showed that TRIM21 functions as a tumor suppressor in breast cancer to regulate the proliferation, invasiveness, EMT, and stem-like properties.

### MTA1 promotes the proliferation, invasion, and cancer stemness of breast cancer cells through hindering MTA3/TRIM21 expression

The EdU assays in breast cancer cells showed that the decrease in proliferation mediated by MTA1 knockdown was reversed by MTA3 or TRIM21 knockdown (Fig. 5H). MTA1-overexpression promoted the invasiveness of breast cancer cells, while overexpression of MTA3 or TRIM21 restored it to normal levels. MTA1 loss-of-function in MDA-MB-231 cells decreased invasiveness, which was partly restored through co-knockdown of MTA3 or TRIM21(Fig. 5I, J). These results revealed that MTA1 promotes the proliferation, invasiveness, and stemness of breast cancer cells, in part by inhibiting MTA3 expression.

### TRIM21 regulates MTA1 degradation

**Fig. 6:**
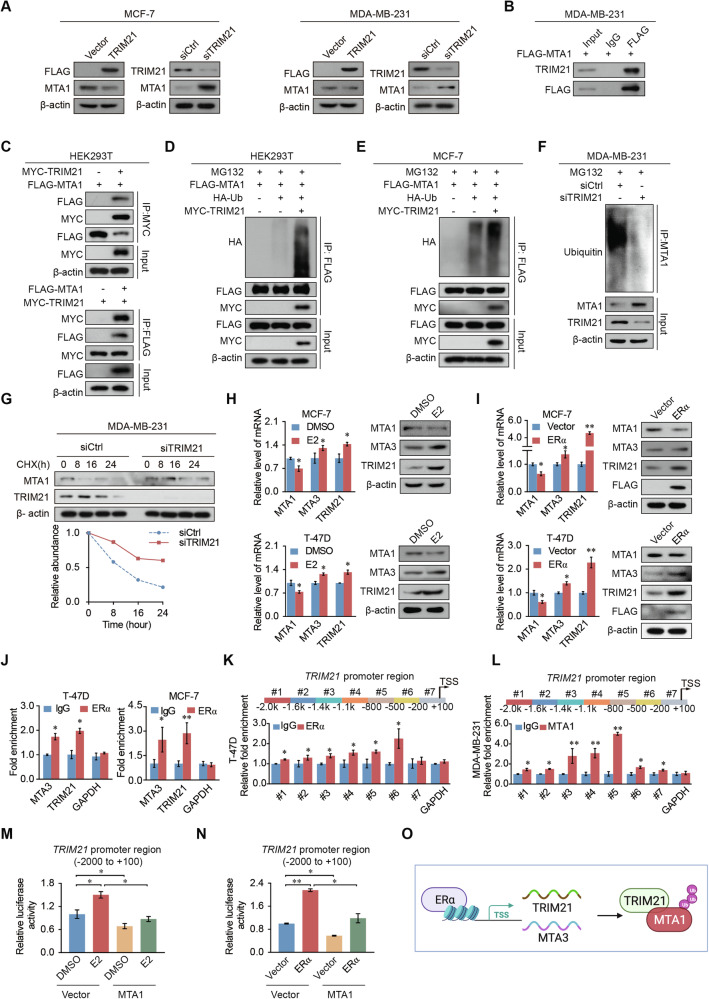
MTA1 expression is regulated by TRIM21-mediated ubiquitination, and ERα promotes TRIM21 transcriptional activation. **A** TRIM21 hinders MTA1 expression in MCF-7 and MDA-MB-231 cells. The protein expression of MTA1 was detected after overexpression or knockdown of TRIM21 cells. **B** MTA1 interacts with TRIM21. Whole cell lysates of MDA-MB-231 cells overexpressing MTA1 were immunoprecipitated with control IgG or FLAG antibodies, followed by western blotting with the indicated antibodies. **C** HEK293T cells were transfected with FLAG-MTA1 alone or together with MYC-TRIM21 for 36 h, then immunoprecipitated with anti-FLAG and anti-MYC antibodies, followed by western blotting. **D, E** HEK293T (**D**) and MCF-7 (**E**) cells were transfected with FLAG-tagged MTA1, HA-tagged Ub, and MYC-tagged TRIM21 alone or together for 24 h and treated with 20 μM MG132 for 8 h. The cell lysate was immunoprecipitated with an anti-FLAG antibody and western blotting was performed with the corresponding antibody. **F** TRIM21 affects the protein stability of MTA1. MDA-MB-231 cells were transfected with control or TRIM21 siRNA for 40 h and then treated with MG132 (20 μM) for 8 h. Cell lysates were immunoprecipitated with MTA1 antibody, and western blotting was performed using the corresponding antibodies. **G** Western blotting revealed that TRIM21 affected the protein stability of MTA1. MDA-MB-231 cells transfected with control or TRIM21 siRNA were treated with CHX (50 μg/ml) for 0 h, 8 h, 16 h, and 24 h. **H**, **I** qRT-PCR and western blot results show that E2 treatment (**H**) or FLAG-ERα transfection (**I**) promotes the expression of MTA3 and TRIM21 and inhibits the expression of MTA1 in MCF-7 and T-47D cells. **J** ChIP assays showed that ER*α* binds in the promoter region of MTA3 and TRIM21. **K**, **L** Primer pairs #1–7 were synthesized to cover the promoter region of *TRIM21*, and a qChIP-based promoter walking experiment was performed using ERα antibody (**K**) or MTA1 (**L**) antibodies. **M**, **N** Luciferase activity of TRIM21 promoter (−2000 to +100) reporters in MDA-MB-231 cells with E2 treatment (**M**) or FLAG-ERα (**N**) transfection after transfection with vector and MTA1. **O** The proposed regulatory mechanisms of ERα, MTA3, MTA1, and TRIM21. Error bars represent the mean ± SD in **H**–**N**. *p < 0.05, **p < 0.01; two-tailed unpaired *t*-test.

Previous studies have shown that TRIM21 is an E3 ligase [21]. To unravel the mechanism underlying the relationship of TRIM21 and MTA1, we further explored whether TRIM21 could affect the ubiquitination and protein stability of MTA1. Western blotting showed that overexpression of TRIM21 in breast cancer cells caused a diminishing in the protein level of MTA1, whereas TRIM21 knockdown increased it (Fig. 6A). Immunoprecipitation (IP) assays revealed that TRIM21 co-immunoprecipitated with MTA1 (Fig. 6B and Supplementary Fig. 6A). Further IP assays in HEK293T cells showed that FLAG-tagged MTA1 was pulled down by MYC-tagged TRIM21, and vice versa (Fig. 6C). The overexpression of wild-type TRIM21 with HA-ubiquitin and FLAG-MTA1 markedly promoted MTA1 ubiquitination (Fig. 6D, E). In contrast, TRIM21 knockdown inhibited MTA1 ubiquitination (Fig. 6F and Supplementary Fig. 6B). The half-life of MTA1 was prolonged by knocking down TRIM21 and inhibiting protein synthesis by cycloheximide (CHX) treatment (Fig. 6G). These results indicate that TRIM21 regulates the protein stability of MTA1 by affecting MTA1 ubiquitination.

### Estrogen receptor α and MTA1 regulate TRIM21 transcription

Our previous results indicated MTA1 and estrogen receptor α (ERα) mutually inhibit each other’s expression [22, 23]. TRIM21 is downregulated by MTA1 and plays a vital role in breast cancer. Therefore, the relationship between TRIM21 and ERα makes us curious to further explore. The expression of TRIM21 and MTA3 was diminished after E2 treatment, whereas MTA1 expression was upregulated by E2 treatment (Fig. 6H). Transfection of FLAG-ERα contributed to the upregulated expression of TRIM21 and MTA3 and led to the decreased expression of MTA1 in breast cancer cells (Fig. 6I). ChIP experiments showed that ERα was enriched in the promoter regions of *TRIM21* and *MTA3* (Fig. 6J). qChIP-dependent promoter walk experiments showed that, in breast cancer cells, ERα was mainly enriched in the −800 to −200 section of the *TRIM21* promoter (Fig. 6K and Supplementary Fig. 6C), MTA1 was mainly enriched in the −1 400 to −500 region of the *TRIM21* promoter (Fig. 6K). Furthermore, the luciferase activity of the *TRIM21* reporter containing its promoter (−2000 to +100) was significantly activated by E2 treatment or transfection of FLAG-ERα, and this activation is inhibited when MTA1 is overexpressed (Fig. 6M, N). TRIM21 or MTA3 expression had good predictive ability for the survival of patients with ER-positive breast cancer, however, no significant difference was observed in survival in patients with ER-negative breast cancer (Supplementary Fig. 6D, E). These results indicated that MTA1 and ERα can bind to the promoter of *TRIM21*. The promoter region of *TRIM21* binding to MTA1 led to a reduction of its expression, whereas ERα binding transcriptionally activated it expression (Fig. 6O).

### MTA1 upregulation is a marker for poor prognosis, whereas MTA3 and TRIM21 are correlated with good prognosis in multiple cancers

**Fig. 7:**
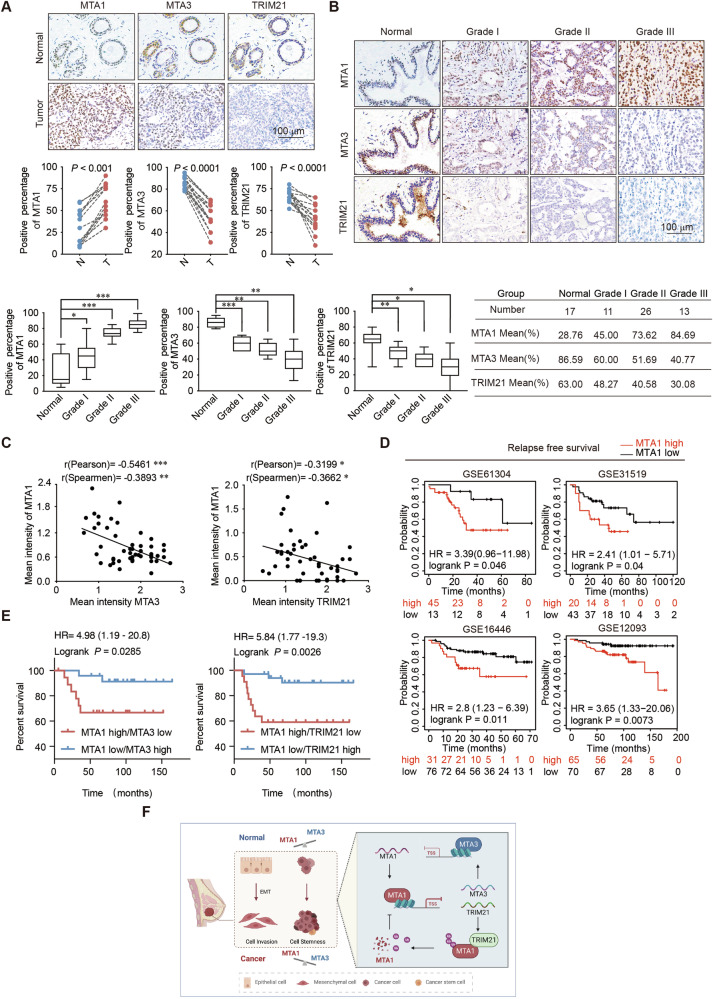
MTA1 is upregulated in breast cancer and is a potential cancer biomarker. **A** Immunohistochemical analysis of MTA1, MTA3, and TRIM21 in breast cancer and adjacent normal tissues (*n* = 10). N adjacent normal tissue, T tumor tissue. **B** Immunohistochemical staining of normal breast tissue and tumors (histological grades I, II, and III) for MTA1. Representative images of each grade are shown. The percentage of positively stained nuclei (percentage) in the grouped samples was analyzed using a two-tailed unpaired *t*-test. **C** Correlation analysis shows negative correlations between the expression levels of MTA1 and MTA3, MTA1 and TRIM21 were negatively correlated in expression. **D** RFS survival analysis showed that breast cancer patients with high MTA1 expression have a good prognosis. **E** Survival analysis of clinical data on the relationship between survival time and MTA1/MTA3 and MTA1/TRIM21 expression signatures in breast cancer. Survival curves were calculated using the Kaplan–Meier curves. **F** Schematic representation of regulatory mechanisms for MTA1, MTA3 and TRIM21 in promoting breast cancer development. Error bars represent the minimum to maximum in **B**. *p < 0.05, **p < 0.01, ***p < 0.001; two-tailed unpaired *t*-test.

To further explore the predictive performance of MTA1, MTA3, and TRIM21 in the prognosis of patients with breast cancer, we performed immunohistochemical staining. The findings indicated that MTA1 expression was significantly upregulated in breast cancer tissues compared to that in normal tissues, whereas MTA3 and TRIM21 were the opposite. MTA1 expression was positively correlated with cancer histological grade, whereas those of MTA3 and TRIM21 were the opposite (Fig. 7A, B). MTA1 expression negatively correlated with MTA3 and TRIM21 expression (Fig. 7C). Kaplan–Meier survival analysis displayed that higher MTA1 expression level is detrimental to RFS and OS in breast cancer patients (Fig. 7D and Supplementary Fig. 7A). This also applies to cervical squamous cell carcinoma, liver hepatocellular carcinoma, and sarcoma (Supplementary Fig. 7B, C).

Analysis of the GSE58812 dataset found that the stratification of patients based on the converse expression of MTA1/MTA3 or MTA1/TRIM21 improved the predictive power of MTA1 (Fig. 7E). These findings suggest that MTA1, MTA3, and TRIM21 are potential biomarkers for breast cancer.

## Discussion

This study revealed negative regulatory relationships between MTA1 and MTA3/TRIM21. The crosstalk between MTA1 and MTA3 regulates the malignancy of breast cancer cells, including proliferation, invasion, EMT, and stemness. Additionally, this study also suggests that TRIM21 influences the stability of MTA1. The regulatory mechanisms of MTA1, MTA3, and TRIM21 affecting breast cancer progression via EMT and stem cell properties is shown in Fig. 7F.

Cancer stem cells with unlimited growth potential to increase in value are closely associated with the processes of tumor metastasis, recurrence, and drug resistance [24]. Breast cancer stem cells are involved in multistep of breast cancer development containing invasion and metastasis [25]. Cancer stem cell production is mediated by the EMT, which promotes cellular depolarization and self-renewal to promote tumorigenesis [4, 26]. Our study identified multiple regulators of breast cancer stemness and showed that MTA1 influences the cancer stem-like state by interacting with EMT and inhibiting the expression of MTA3 and TRIM21.

Studies have shown that the expression of MTA1 is regulated by multiple genes, including MUC1-C/MYC [27, 28]. MTA1 is ubiquitinated by the E3 ubiquitin ligase COP1 [29, 30]. MTA1 expression is also inhibited by stilbenes, thereby disrupting the MTA1/histone deacetylase complex [31]. In this study, MTA3 and TRIM21 were found to restrict the expression of MTA1 in different ways. MTA3 formed a transcriptional repression complex in the promoter region of MTA1, hindering the transcription of MTA1. TRIM21 affected the protein stability of MTA1 by ubiquitinating MTA1.

Additionally, the transcription of MTA1 is also activated by the clock circadian regulator basic helix-loop-helix arnt like 1 (CLOCK-BMAL1) heterodimer [32]. Furthermore, a bacterial lipopolysaccharide has been shown to activate MTA1, leading to increased expression of transglutaminase 2, thereby modulating the inflammatory response [33]. Our findings showed that the hyperactivation of MTA1 in breast cancer cells drives EMT and cancer stemness.

The NuRD complex is an important transcriptional regulator with a crucial role in tumors via transcriptional repression [34]. The NuRD (MTA1) complex promotes EMT through transcriptional repression of peroxisome proliferator-activated receptor α (*PPARα*) and superoxide dismutase 2 (*SOD2*) in breast cancer [35]. MTA1 is involved in a variety of malignancies by regulating the EMT process, involving multiple pathways [36–42]. The NuRD (MTA1) complex influences cancer EMT by modulating E-cadherin expression, thereby promoting cancer invasiveness and metastasis [17]. Our qChIP results showed that MTA1 transcriptionally regulates tumor suppressors, including MTA3 and TRIM21. Our previous study showed that NuRD (MTA1) and NuRD (MTA3) form an antagonism that potentially regulates breast cancer development and progression [16, 23]. In this study, we discovered that MTA1 is significantly enriched in the promoter region of *MTA3* to inhibit the transcription of *MTA1*, whereas MTA3 is relatively enriched in the *MTA1* promoter to hinder its transcription.

TRIM21 consists of a zinc finger coiled-coil, and RING structural domains for E3 ubiquitin ligation [43]. Meanwhile, TRIM21 inhibits the transcription of runx family transcription factor 2 (RUNX2) by regulating the degradation of set domain containing 7/9 (SET7/9), thereby inhibiting the malignant phenotype in breast cancer [44]. In breast cancer cells, TRIM21 regulates the EMT process by affecting the proteasomal degradation of SNAI1. Low expression of TRIM21 positively correlates with poor prognosis of patients with breast cancer [45]. TRIM21-mediated degradation of interferon gamma inducible protein 16 (IFI16) and hu-antigen R (HuR) inhibits radiation therapy resistance in stemness of gliomas [46]. TRIM21 inhibits the stemness of gastric cancer cells and functions as a sensitizer to apatinib treatment [47]. It can be affected by both SUMOylation and cop9 signalosome subunit 6 (CSN6)- mediated ubiquitination in colon cancer [48, 49]. Our results revealed that TRIM21 hindered proliferation, invasion, EMT, and stemness in breast cancer.

TRIM21 regulates the ubiquitination of several proteins [44, 50–52]. This study is the first to demonstrate that TRIM21 interacts with MTA1 to regulate the ubiquitination of its proteins and attenuate the protein expression level of MTA1. TRIM21 is involved in the progression of cancers, mainly metabolism, immune regulation, and cancer therapy [43, 53–55]. TRIM21 mediates the degradation of branched-chain amino acid transaminase 2 (BCAT2) to inhibit the formation and progression of pancreatic ductal adenocarcinoma [56]. TRIM21 mediates the degradation of the autophagic lysosomal pathway of cyclin-dependent kinase 2 (CDK2) to inhibit the proliferation of acute myeloid leukemia [57]. Our findings revealed that TRIM21 affects the stability of MTA1 to hinder breast cancer proliferation, invasion, EMT, and cancer stemness and synergizes with MTA3 to maintain epithelial homeostasis.

In conclusion, our research provides mechanistic understandings that the negative feedback loops between MTA1 and MTA3, MTA1, and TRIM21 in response to estrogen are initiated to regulate cancer stem cell fate and EMT in breast cancer. MTA1 exerts negative regulation through transcriptional inhibition of MTA3 and TRIM21. Conversely, MTA3 inhibits MTA1 mRNA expression through transcriptional regulation, and TRIM21 influences the stability of MTA1. Our results add to sights of the complexity of EMT and stemness regulatory networks and suggest that MTA1 is a potential biomarker for breast cancer diagnosis and prospective therapeutic target. MTA3 and TRIM21 are potential prognostic biomarkers for breast cancer.

## Materials and methods

### Antibodies and reagents

Antibodies were purchased MTA1(5647, 5646), E-Cadherin (3195), α-N-Cadherin (3240), γ-Catenin (2309), N-Cadherin (13116), Vimentin (5741), NANOG (4903), SOX2 (3579), c-MYC (18583) from Cell Signaling Technology (Danvers, MA, USA); HA (ab9110) and OCT4 (ab19857) from Abcam (Cambridge, UK); fibronectin (F3648), FLAG (F1408) from Sigma-Aldrich (St. Louis, MO, USA); and MTA3 (sc-81325), TRIM21 (sc-25351) from Santa Cruz Biotechnology (Dallas, TX, USA) and. The short interfering RNAs (siRNAs) and small hairpin RNAs (shRNAs) were obtained from GenePharma (Shanghai, China). MG132 and cycloheximide (CHX) were bought from Sigma-Aldrich. Matrigel (354248 & 354262) was purchased from Corning Inc. (NY, USA).

### Cell culture and transfection

The American Type Culture Collection (ATCC, Manassas, VA, USA) provided the cell lines including MDA-MB-231, MCF-7, HEK293T, T-47D, BT-474, and SK-BR-3. The Asterand Bioscience (Detroit, Michigan, USA) provided SUM159. Dulbecco’s modified Eagle’s medium (DMEM) (Biological Industries, Kibbutz Beit-Haemek, Israel) supplemented with 10% fetal bovine serum (FBS) (Biological Industries, Kibbutz Beit-Haemek, Israel) and 1% penicillin–streptomycin (Thermo Fisher Scientific, Waltham, MA, USA) was used to cultivate MDA-MB-231, MCF-7, and HEK293T cells. T-47D, BT-474, SK-BR-3, and SUM159 cells were maintained at 37 °C and 5% CO_2_ in RPMI 1640 medium with 10% FBS and 1% penicillin–streptomycin. Before T-47D and MCF-7 were treated with 17*β*-estradiol (E2) or FLAG-ERα transfection, they were cultured using phenol red-free medium over 72 h. Transfections were performed using polyethyleneimine (Polysciences, Warrington, PA, USA), TurboFect Transfection Reagent (Thermo Fisher Scientific), and Lipofectamine RNAiMAX Reagent (Thermo Fisher Scientific), as per the requirements of the manufacturer. Three or more repetitions of each experiment were conducted. Supplementary Tables 1 and 2, respectively, contain the siRNA and shRNA sequences used in this study.

### 5-ethynyl-2′-deoxyuridine assay

Ten to twenty unfilled Petri dish wells were injected with cells. Following cell apposition, the cells underwent the EdU assay in accordance with the 5-ethynyl-2′-deoxyuridine assay (EdU) kit’s instructions (Ribobio, Guangzhou, China). Using a fluorescent microscope, pictures of the cells were taken.

### Wound-healing assay

Cells were inoculated in culture dishes and cultured with DMEM medium with 10% FBS to 90% confluence, after which scratches were produced using a 200 μL pipette tip, followed by 3 gentle rinses with phosphate-buffered saline (PBS). After that, the cells were incubated for 36 hours at 37 °C in new media devoid of serum. Under a microscope, cells in a 6-well plate were captured on camera.

### Cell invasion assay

Matrigel™ was considered for covering Transwell chambers. The Transwell’s upper chamber was filled with cells cultured in a serum-free medium. The lower chamber contained medium containing 10% FBS. After the removal of upper chamber, the cells were stored for 10 minutes with 1 mL methanol and stained with 1 mL crystal violet for ten minutes. After the cells were fixed and stained, they were photographed using microscope.

### Spheroid-forming assay

Trypsin treatment was administered to cells in the logarithmic growth phase, followed by a 5-min centrifugation at 200×*g* and resuspension in MammoCult™ medium (StemCell Technologies, Vancouver, BC, Canada, #05620). Cells were incubated in low adsorption 6-well plates containing MammoCult™ medium at 37 °C with 5% CO_2_. Every 3 days, the medium was replaced, and on day fifteen, the outcomes were presented under a microscope.

### Mouse xenograft model

After being gathered, cells in the logarithmic growth phase underwent 3 PBS washes. After that, the cells were resuspended in PBS along with Matrigel and injected into the fourth pair of mammary fat pads of 4-week-old non-obese diabetic/server combined immune-deficiency (NOD/SCID) mice, which are randomly numbered and grouped. The size of tumors was monitored weekly. Length × width^2^/2 is the formula to calculate tumor volume. The frequency of cancer stem cells was detected using the software Extreme Limiting Dilution Analysis (ELDA) (https://bioinf.wehi.edu.au/software/elda/index.html).

### Flow cytometry

After being resuspended, the cells were mixed with 1% bovine serum albumin (Solarbio, Beijing, China) in phosphate-buffered saline (PBS), along with the addition of the appropriate antibody following instructions before incubation at 4 °C for 45 minutes, sheltered from light. After that, the cells were cleaned 3 times using PBS containing 1% BSA. BD BioSciences, Franklin Lakes, NJ, USA, provided the FlowJo software 10.4.0, which was used for data analysis and cell assay.

### Real-time quantitative PCR

Based on the instructions of manufacturer, cellular RNA was isolated with the help of RNA-Quick Purification Kit (Esunbio, Shanghai, China). PrimeScriptTM RT Master Mix (TaKaRa Bio, Kusatsu, Shiga, Japan) was used to produce cDNA. Real-time quantitative transcripts were assayed using ABI QuantStudio5 and analyzed using the comparative Ct method, with GAPDH as an internal reference. Supplementary Table 3 contains a list of primer sequences utilized in this investigation.

### Immunoprecipitation and western blotting

Cells were prepared by washing 3 times by using PBS at 4 °C and collecting in tube by centrifugation at 200 × g for 5 min at 4 °C. The cells were lysed with lysis buffer (0.005 M Tris-HCl, pH 7.5, 0.15 M NaCl, 1 mM EDTA, 0.5% NP-40, 0.25% sodium deoxycholate, with adding protease inhibitor cocktail) for 30 min at 4 °C, followed by centrifugation at 12 000 × g for 10 min. The 500 μg proteins was added to the appropriate (2–3 µg) antibody and incubated overnight at 4 °C. Protein A/G Sepharose CL-4B (Life Technologies, Carlsbad, CA, USA) beads were then included and the samples were gestated for 1–2 h at 4 °C. After washing the solution six times, the beads were mixed with 1 × loading buffer boiling 10 min at 95 °C to separate the proteins. Western blotting was then performed. 10% Sodium dodecyl sulfate - polyacrylamide gel electrophoresis (SDS-PAGE) electrophoresis was conducted, after which gel was transferred onto polyvinylidene difluoride (PVDF) membrane. Primary antibody incubation and secondary antibody incubation were performed next. Enhanced chemiluminescence (ECL System, Thermo Scientific) based on the instructions by the manufacturers. Raw images are shown in original western blots–Supplementary file 1.

### Chromatin immunoprecipitation and quantitative ChIP

ChIP experiments were accomplished using breast cancer cells. Cells with 100% confluent 15 cm cell culture dish were prepared to wash 3 times using room temperature PBS at 37 °C. Next, cells were incubated with 1% formaldehyde diluted with PBS at 37 °C. The lysis solution was then added after the cells had been cleaned 3 times with 4 °C PBS. The supernatant was obtained by centrifugation at 2 000 × g for 10 min. Next, the samples were incubated overnight with 2–3 μg of antibody at 4 °C. The samples were incubated with Protein A/G Sepharose CL-4B beads (Life Technologies, Carlsbad, CA, USA) 4 h at 4 °C before washing five times using washing buffers at 4 °C. The DNA was then eluted with PCR Purification Kit (Macherey-Nagel, Duren, Germany) and used for subsequent PCR or RT-qPCR experiments. In Supplementary Tables 4 and 5, the primer sequences used in this investigation are displayed.

### Luciferase reporter assay

48 hours after transfection, cells were lysed using the lysis solution after being transfected with the matching plasmids. The luciferase activity test was performed on the lysates in accordance with the Luciferase Reporter Kit’s (Promega, Madison, WI, USA) instructions.

### Tissue specimens and immunohistochemistry

The samples were fixed overnight in 4% paraformaldehyde before embedding in paraffin and cutting 8 µm sections. The sections were dewaxed, and the primary antibody added before incubating overnight at 4 °C. After 2 h of secondary antibody incubation, the samples were stained with diaminobenzidine. The samples were obtained in compliance with the ethical protocol was authorized by the Chinese Academy of Medical Sciences’ Cancer Hospital Ethics Committee, and all patients provided informed consent.

### Bioinformatical and statistical analysis

Differential genes obtained by RNA-seq were used for KEGG analysis by using the DAVID (Database for Annotation, Visualization, and Integrated Discovery) online (https://david.ncifcrf.gov/). Gene expression profiles from RNA sequencing were used for Gene Set Enrichment Analysis (GSEA) analysis utilizing the GSEA app. The findings were assessed with the help of SPSS version 22 (IBM Corp., Armonk, NY, USA) and GraphPad Prism 8 (GraphPad Software, Boston, MA, USA) and are presented as mean ± SD. Tumor datasets were downloaded from https://www.ncbi.nlm.nih.gov/geo/, and the GSE numbers are shown in the text. Kaplan–Meier survival analyses were performed using http://kmplot.com/analysis/.

### Supplementary information

Supplementary Figures and Tables

Original western blots

## Data Availability

The datasets used in the current study are available from the corresponding author on reasonable request. All data generated or analyzed during this study are included in this article and its supplementary information files. The full length uncropped original western blots were uploaded as original western blots.
