# Supplementary material for: The feedback loop between MTA1 and MTA3/TRIM21 modulates stemness of breast cancer in response to estrogen
Source: Cell Death Dis. 2024 Aug 17;15(8):597. doi: 10.1038/s41419-024-06942-w (PMC11330498; doi:10.1038/s41419-024-06942-w)
Supplement: Supplementary file 1 — Supplementary Figures and Tables [file 41419_2024_6942_MOESM1_ESM.docx]

**This file includes:**

Supplementary Figures 1 to 7

Supplementary Tables 1 to 5

**Supplementary Figures and Figure Legends
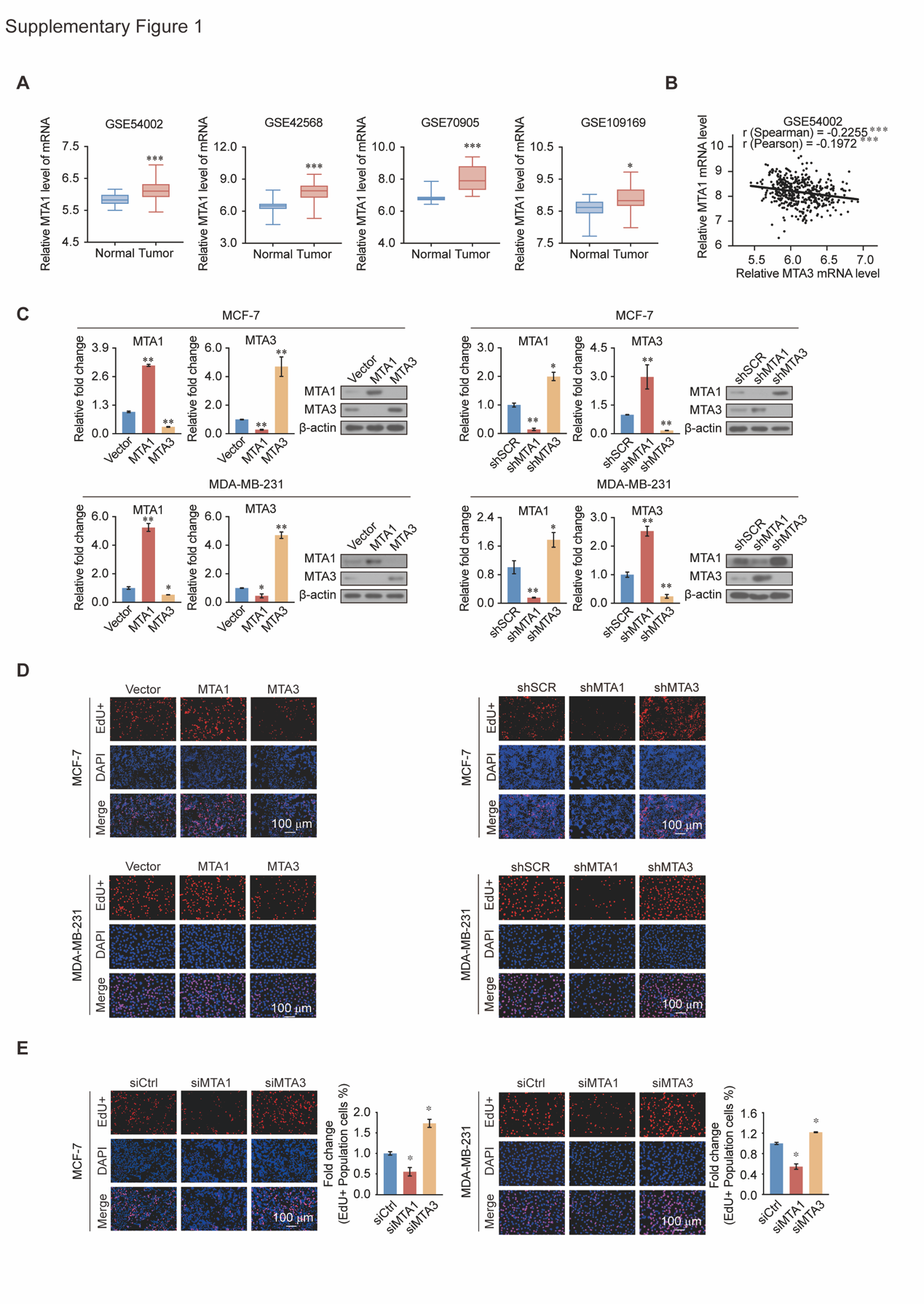
**

**Supplementary Figure 1 The expression of MTA1 is increased in breast cancer and positively with a poor survival.**

**A** Gene Expression Omnibus dataset analysis showed that an increase of MTA1 expression in breast cancer with the comparison of normal tissues using. **B** GSE54002 relevance analysis indicated a negative correlation between MTA1 expression and MTA3 expression in breast cancer. **C** MCF-7 and MDA-MB-231 cells were infected with lentivirus overexpression (vector, FLAG-MTA1 and FLAG-MTA3) or knocked down (shSCR, shMTA1, shMTA3). MTA1 and MTA3 expression was detected by RT-qPCR and Western blotting. **D** EdU images were performed in MCF-7 and MDA-MB-231 cells transfected with vector, MTA1, MTA3, shSCR, shMTA1, or shMTA3. **E** EdU assays in MCF-7 and MDA-MB-231 cells transfected with siCtrl, siMTA1, and siMTA3. siCtrl, siControl. Error bars represent the min to max in **A** and the mean ± SD in **C** and **E**. ∗*p* < 0.05, ∗∗*p* < 0.01, ∗∗∗*p* < 0.001; two-tailed unpaired t-test.

**
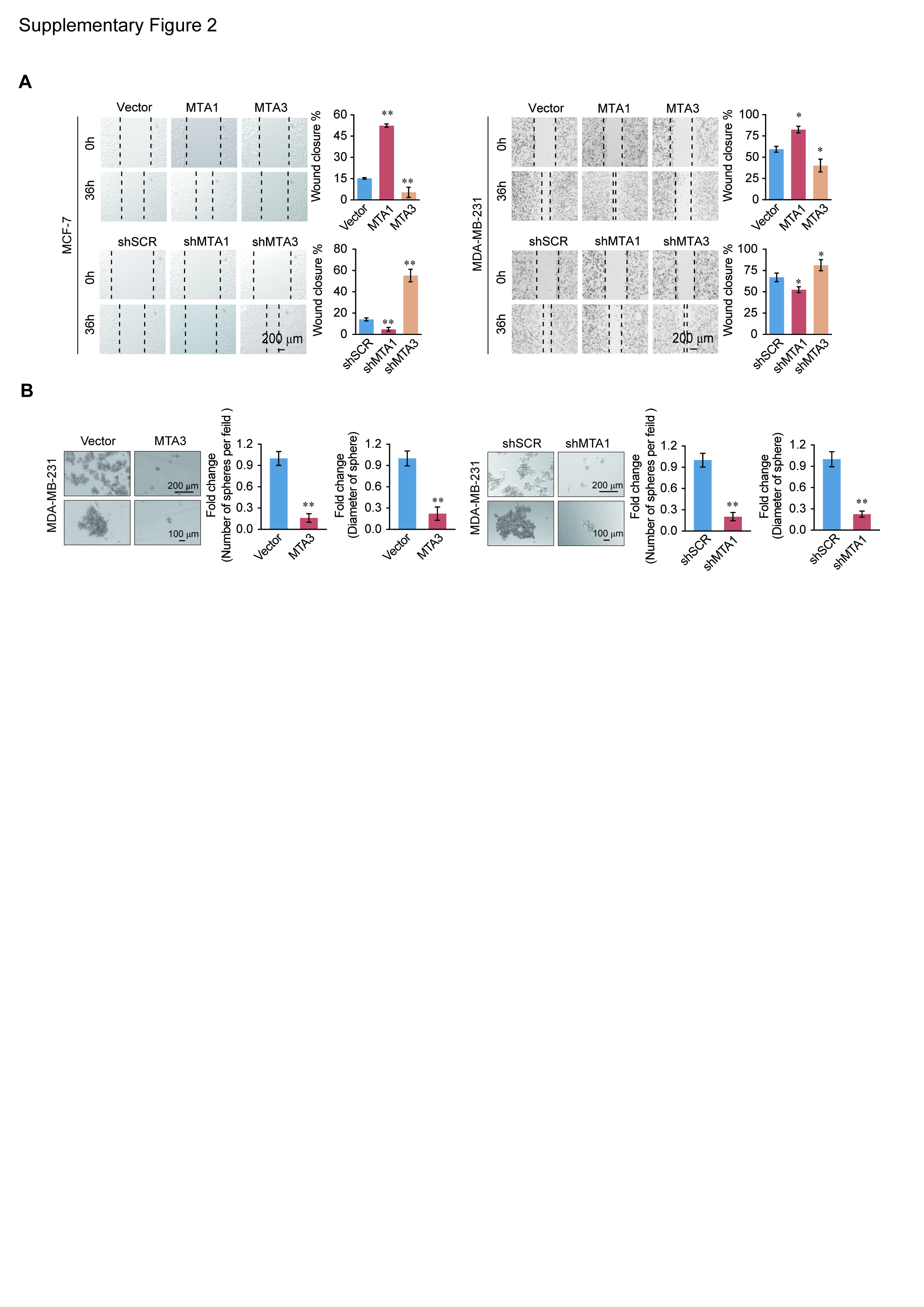
**

**Supplementary Figure 2 MTA1 promotes the migration and spheroid formation of breast cancer cells.**

**A** Wound healing experiments were performed in in MCF-7 and MDA-MB-231 cells transfected with vector, MTA1, or MTA3, or infected with lentiviruses (shSCR, shMTA1 or shMTA3). **B** Representative images of spheroids formation in suspension culture for 15 days MTA3 overexpression and MTA1 knockdown in MDA-MB-231 cells. The number of spheres per field and the diameter of the spheres were statistically analyzed. Error bars represent the mean ± SD in **A** and **B**. ∗*p* < 0.05, ∗∗*p* < 0.01; two-tailed unpaired t-test.


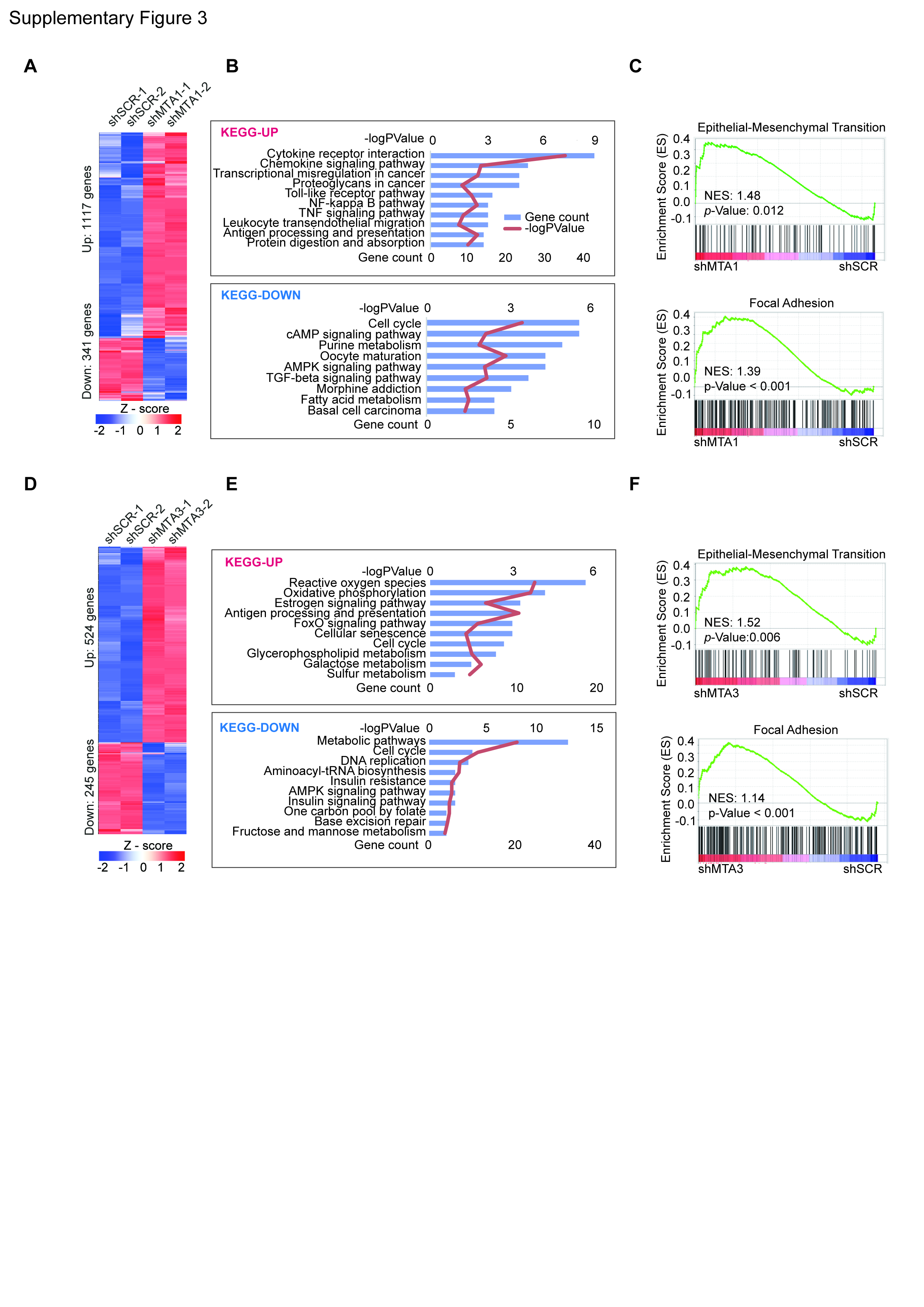


**Supplementary Figure 3 MTA1 regulates tumor suppressor genes expression and participates in EMT- and stemness- related signaling pathway.**

**A** Heatmap of differentially expressed genes ( |Log2 fold-change| >1, *p* < 0.05) in MCF-7 cells stably transfected with shSCR and shMTA1. **B** The bar chart on the right shows the KEGG enrichment analysis of differentially expressed genes using DAVID software (<https://david.ncifcrf.gov/>). **C** Gene set enrichment analysis plot of EMT in shSCR and shMTA1. FDR, false discovery rate; NES, normalized enrichment score. **D** Heatmap of differentially expressed genes (|Log2 fold-change| > 0.5, *p* < 0.05) in MCF-7 cells stably transfected with shSCR and shMTA3. **E** KEGG enrichment analysis of differentially expressed genes is shown on the right via DAVID software. **F** Gene set enrichment analysis (GSEA) plot of EMT in shSCR and shMTA3. FDR, false discovery rate; NES, normalized enrichment score.


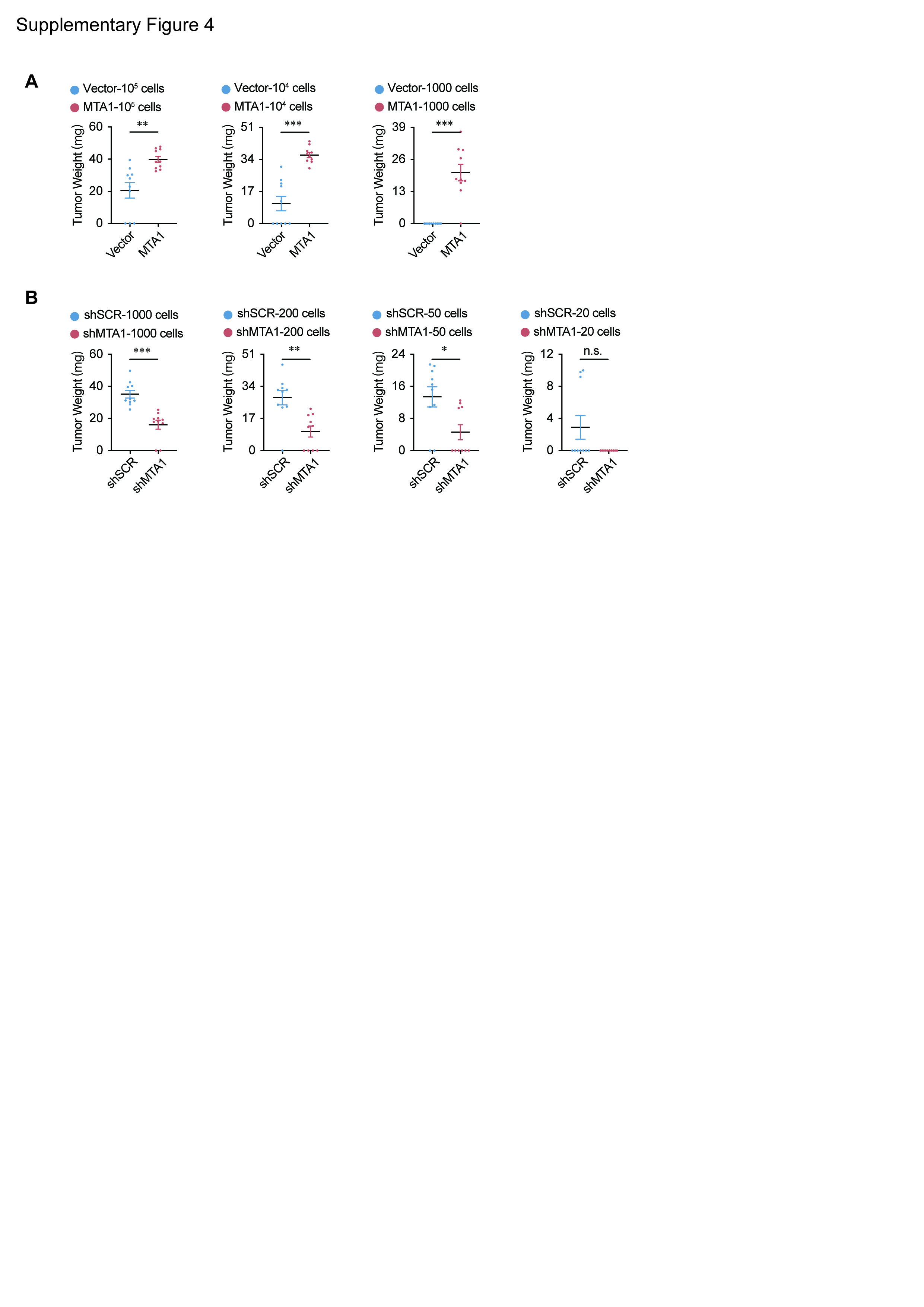


**Supplementary Figure 4 MTA1 promote the frequency of cancer stem cell in mouse xenograft models.**

**A** Tumor weight analysis demonstrated heavier tumors in the MTA1 overexpression group with a comparison of control group. **B** Tumor weight of MTA1 knockdown mouse group and control mouse groups. Error bars represent the mean ± SEM in **A** and **B**. ∗*p* < 0.05, ∗∗*p* < 0.01, ∗∗∗*p* < 0.001; two-tailed unpaired t-test.


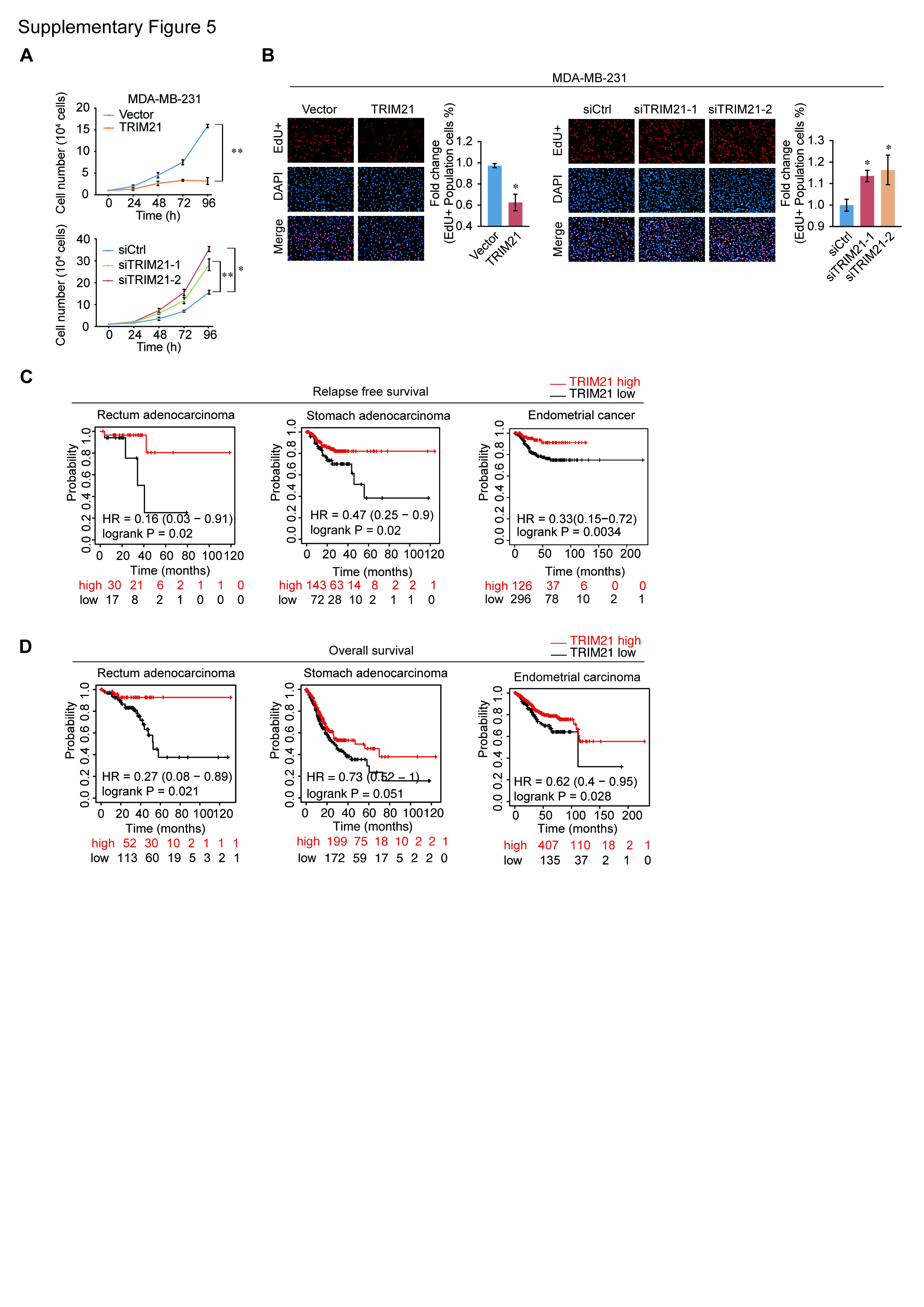


**Supplementary Figure 5 TRIM21 blocks the proliferation in MDA-MB-231 cells and is a potential cancer biomarker.**

**A** Cell count assays indicated that TRIM21 inhibits the growth of MDA-MB-231 cells. **B** EdU assays detected in MDA-MB-231 cells transfected with vector, TRIM21, siCtrl, siTRIM21-1, and siTRIM21-2. siCtrl, siControl. **C** Relapse free survival analysis showing that multiple cancers (rectum adenocarcinoma, stomach adenocarcinoma, and endometrial cancer) that have high expression of TRIM21 have a better survival compared with those that have low expression. **D** Overall survival analysis of TRIM21 high and low expression groups in multiple cancers (rectum adenocarcinoma, stomach adenocarcinoma, and endometrial cancer). Error bars represent the mean ± SD in **A** and **B**. ∗*p* < 0.05, ∗∗*p* < 0.01; two-tailed unpaired t-test.


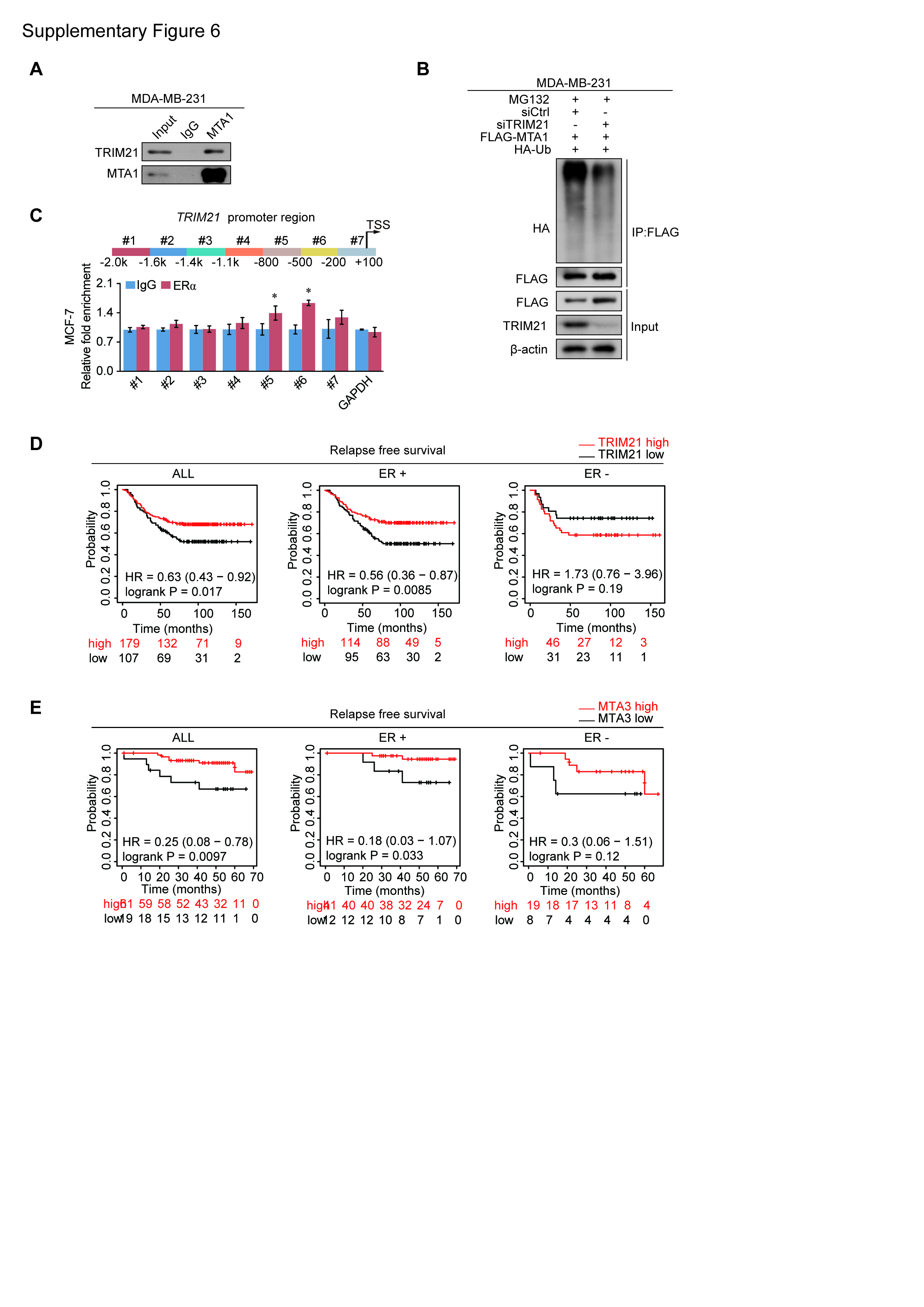


**Supplementary Figure 6 Ubiquitination of MTA1 is regulated by TRIM21, whose expression is activated through ER**α**.**

**A** MTA1 interacts with TRIM21. Whole cell lysates of MDA-MB-231 cells were immunoprecipitated with control IgG or MTA1 antibodies, followed by western blotting with the indicated antibodies. **B** MDA-MB-231 cells transfected with control siRNA or TRIM21 siRNA were then transfected with HA-tagged Ub and FLAG-tagged MTA1 plasmids for 40 hours and treated with 20 μM MG132 for 8 hours. Cell lysates were immunoprecipitated with FLAG antibody, followed by western blotting with the corresponding antibody. **C** Primer pairs #1–7 were synthesized to cover the promoter region of *TRIM21*, and a qChIP-based promoter walking experiment was performed using MCF-7 cells and ERα antibody. **D – E** Relapse free survival analysis (**D**) and overall survival analysis (**E**) of TRIM21 high and low expression in breast cancer. Error bars represent the mean ± SD in **C**. ∗*p* < 0.05, ∗∗*p* < 0.01; two-tailed unpaired t-test.


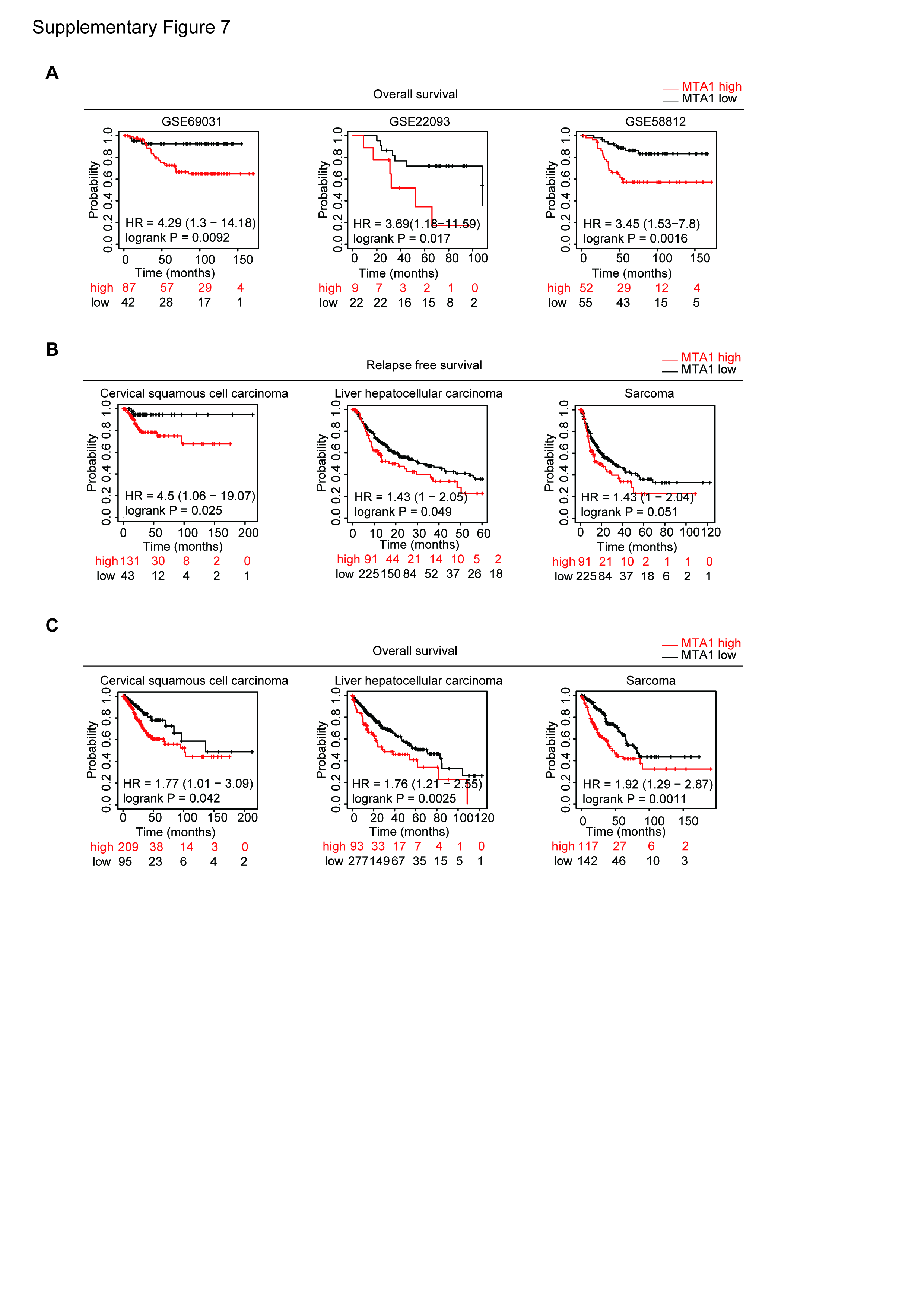


**Supplementary Figure 7 MTA1 is a potential cancer biomarker in multi cancers.**

**A** Kaplan–Meier overall survival analysis was performed to determine on the relationship between survival time and MTA1 expression in breast cancer. **B – C** Kaplan–Meier relapse free survival **(B)** and overall survival **(C)** was performed to determine on the relationship between survival time and MTA1 expression in cervical squamous cell carcinoma, liver hepatocellular carcinoma, and sarcoma.

**Supplemental Tables**

**Supplemental Table 1:**

The siRNA used in this paper were listed as follows:

| ID | Sense（5'-3'） | Antisense（5'-3'） |
| --- | --- | --- |
| siControl | UUCUCCGAACGUGUCACGUTT | ACGUGACACGUUCGGAGAATT |
| siMTA1 | CUAACUUAUUCCGAGAAUGTT | CAUUCUCGGAAUAAGUUAGCC |
| siMTA3 | UUUCUUCCUUCAUACUACATT | UGUAGUAUGAAGGAAGAAAGC |
| siTRIM21-1 | AGAAGUUGGAAGUGGAAAUTT | AUUUCCACUUCCAACUUCUCA |
| siTRIM21-2 | GACUUCACCUGUUCUGUGATT | UCACAGAACAGGUGAAGUCTT |

**Supplemental Table 2:**

The shRNA used in this paper were listed as follows:

| ID | Sense（5'-3'） |
| --- | --- |
| shSCR | TTCTCCGAACGTGTCACGT |
| shMTA1 | CTAACTTATTCCGAGAATG |
| shMTA3 | GCTTTCTTCCTTCATACTACA |

**Supplemental Table 3:**

The Primers used in quantitative real-time PCR (qPCR) were listed as following:

| Gene | Strand | Sequence |
| --- | --- | --- |
| E-cadherin | F | CATTTCTTGGTCTACGCCTG |
| E-cadherin | R | GAGAGGAGTTGGGAAATGTG |
| α-catennin | F | AGCTGAAAGTTGTGGAAGAT |
| α-catennin | R | CCAACATCTTTCAATTCCTGTTG |
| γ-catennin | F | GGACAAGAACCCAGACTACC |
| γ-catennin | R | GTGGCATCCATGTCATCTCC |
| Fibronectin | F | CCATCCATTGATTTAACCAACTT |
| Fibronectin | R | TACCAGGCAGGAGATTTGTTAA |
| N-cadherin | F | CACTGCTCAGGACCCAGAT |
| N-cadherin | R | TAAGCCGAGTGATGGTCC |
| Vimentin | F | ATTGAGATTGCCACCTACAG |
| Vimentin | R | ATCCAGATTAGTTTCCCTCAG |
| OCT-4 | F | ATCACCCTGGGATATACACAG |
| OCT-4 | R | CTGCTTTGCATATCTCCTGA |
| SOX2 | F | GCCTGGGCGCCGAGTGGA |
| SOX2 | R | GGGCGAGCCGTTCATGTAGGTCTG |
| NANOG | F | TCTGGACACTGGCTGAATCCT |
| NANOG | R | CGCTGATTAGGCTCCAACCAT |
| c-MYC | F | AAACTTGAACAGCTACGGAAC |
| c-MYC | R | ATTTGAGGCAGTTTACATTATGG |
| MTA1 | F | ACGCAACCCTGTCAGTCTG |
| MTA1 | R | GGGCAGGTCCACCATTTCC |
| MTA3 | F | GACTTGACCGATAAGCAGAAACA |
| MTA3 | R | AGGGCAACACTGCACTTTCC |
| TRIM21 | F | GTCCTGGAAAGGAGTGAGTCC |
| TRIM21 | R | CTGAAAGTATCAGCCACGGATT |
| ULK1 | F | AGCACGATTTGGAGGTCGC |
| ULK1 | R | GCCACGATGTTTTCATGTTTCA |
| LIFR | F | TGGAACGACAGGGGTTCAGT |
| LIFR | R | GAGTTGTGTTGTGGGTCACTAA |
| PRKACB | F | CCATGCACGGTTCTATGCAG |
| PRKACB | R | GTCTGTGACCTGGATATAGCCTT |
| TNF | F | CTCTTCTGCCTGCTGCACTTTG |
| TNF | R | ATGGGCTACAGGCTTGTCACTC |
| ATP6V0D2 | F | TCTCACCTATATGACGTGCAGT |
| ATP6V0D2 | R | GGTGGCACTTCCCCAGAATTT |
| BASP1 | F | AGGGGAACCCAAAAAGACTGA |
| BASP1 | R | GGTGTGGAACTAGGCGCTTC |
| DGAT2 | F | ATTGCTGGCTCATCGCTGT |
| DGAT2 | R | GGGAAAGTAGTCTCGAAAGTAGC |
| ITGA7 | F | CAGCGAGTGGACCAGATCC |
| ITGA7 | R | CCAAAGAGGAGGTAGTGGCTATC |
| GAPDH | F | GTCAACGGATTTGGTCGTAT |
| GAPDH | R | GAACATGTAAACCATGTAGTTGA |

**Supplemental Table 4:**

The primers used in ChIP assays were listed as following:

| Gene | Strand | Sequence |
| --- | --- | --- |
| MTA3 | F | CATCCAGGCTGCGGTTGT |
| MTA3 | R | CGTGTCGGTGGTGTTCGT |
| TRIM21 | F | TCCCATGTTTATCGCAGCACT |
| TRIM21 | R | ATTCCCCACTACCCTTCCCA |
| ULK1 | F | CTTTCGAGGCTGGATCCCTAC |
| ULK1 | R | GCGACCTCCAAATCGTGCTT |
| LIFR | F | TGCTGATGCATTCCAGTCATTG |
| LIFR | R | TGTACCTGGAGGGCGATGAA |
| PRKACB | F | TCTGTTCACCATGCTACCCAGTC |
| PRKACB | R | AGATGCCATCCATGTGGTATTCA |
| GAPDH | F | GCCTCCAAACAGCCTTGC |
| GAPDH | R | GCCCTGACTTCCTCCACCT |

**Supplemental Table 5:**

The primers used in qChIP-based promoter walk assays were listed as following:

| ID | Strand | sequencing |
| --- | --- | --- |
| MTA1-1 | F | CACGCCATTCTTCTGCCTCAG |
| MTA1-1 | R | TCGAGACCATCCTGGCTAACAC |
| MTA1-2 | F | GTTCACTGGTCTGGAGTGGG |
| MTA1-2 | R | GTCCTTCTGAGGGAGCACAG |
| MTA1-3 | F | GTCTCCACTCCAGCAGTCAGTT |
| MTA1-3 | R | GAGAACCCAGCCACACCACT |
| MTA1-4 | F | CTGCTGAGGAGACAGAGGTAGG |
| MTA1-4 | R | TGCTGGTGATTGGGAGGGAAG |
| MTA1-5 | F | TGGCTTTCCTCCCAGCACAT |
| MTA1-5 | R | GGACAGACATGGTCAGGATGCT |
| MTA1-6 | F | ACGCTCCGCTCCATGTCCTA |
| MTA1-6 | R | GGTTCCAGGCTGCTGATGTGA |
| MTA1-7 | F | TCCTGGACTCCAGCCCTGTT |
| MTA1-7 | R | TGAACCGTGACCTGGTGAGAAG |
| MTA1-8 | F | AAATGGCCCTTGTGGAGGATCT |
| MTA1-8 | R | TTCCCGGACAGTTCCTCTCG |
| MTA3-1 | F | TAGGGCTGGTTGATGACAAGTT |
| MTA3-1 | R | CTTTCAACCAAAGACGGGTGG |
| MTA3-2 | F | AGGGAGGAGTTTTCTCGGCA |
| MTA3-2 | R | AGTGCTCTTGCCAGGTGTTTA |
| MTA3-3 | F | CCAGAGGAAAGGGCTGATTCAT |
| MTA3-3 | R | CCAGAGATCCCACCAAAAACATT |
| MTA3-4 | F | AGCAGAGAACCCTTGGGAATC |
| MTA3-4 | R | CCATGCAAATAACAGGTGTCTCT |
| MTA3-5 | F | TTCTTTTCTGGGGGCACCTC |
| MTA3-5 | R | TCTTGGTGTGGTGGCTTGTT |
| MTA3-6 | F | TGAGAGTAGGAAGGCCGTGA |
| MTA3-6 | R | GGAACAGGGCCCTACAGTTC |
| MTA3-7 | F | CCCTTGAATCTCACCGCGA |
| MTA3-7 | R | GCGCCAGTCTCAATGCAAAC |
| MTA3-8 | F | CAACATGTACCGGGTCGGAG |
| MTA3-8 | R | GCTCCCGGAACCCGC |
| TRIM21-1 | F | TCCATAAGCACAGGCAACCA |
| TRIM21-1 | R | CCCTTGTCAGATGGATAGTTTGC |
| TRIM21-2 | F | GTGTTCAACACCACTGATCATCA |
| TRIM21-2 | R | TGCCAGCATTTGTTATTGCCT |
| TRIM21-3 | F | TCCCATGTTTATCGCAGCACT |
| TRIM21-3 | R | TGGCTGAATAGTGCTCCATTGT |
| TRIM21-4 | F | CCAAAGGCTGGGAAGGGTAG |
| TRIM21-4 | R | TGACTGTATTCACCTTGTTATGCT |
| TRIM21-5 | F | ACACCTACTATAGACACACAAGATTGA |
| TRIM21-5 | R | ACTGTGTTCTTGTCACTGAGCT |
| TRIM21-6 | F | GGCCACTCTTCTCTGCATCA |
| TRIM21-6 | R | CGGGCTGCTTTCTTTGACAC |
| TRIM21-7 | F | CACGGGCTACTGAGTTTCCA |
| TRIM21-7 | R | GCGGCTGAGAAGAGAAGTCA |
