# Supplementary material for: The feedback loop between MTA1 and MTA3/TRIM21 modulates stemness of breast cancer in response to estrogen
Source: Cell Death Dis. 2024 Aug 17;15(8):597. doi: 10.1038/s41419-024-06942-w (PMC11330498; doi:10.1038/s41419-024-06942-w)

Figure 1E

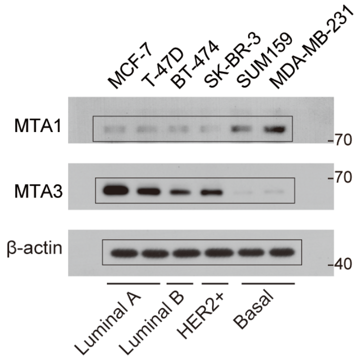
MCF-7
T-47D
BT-474
SK-BR-3
SUM159
MDA-MB-231
MTA1
MTA3
β-actin
70
70
40
Luminal A
Luminal B
HER2+
Basal

Figure 2B

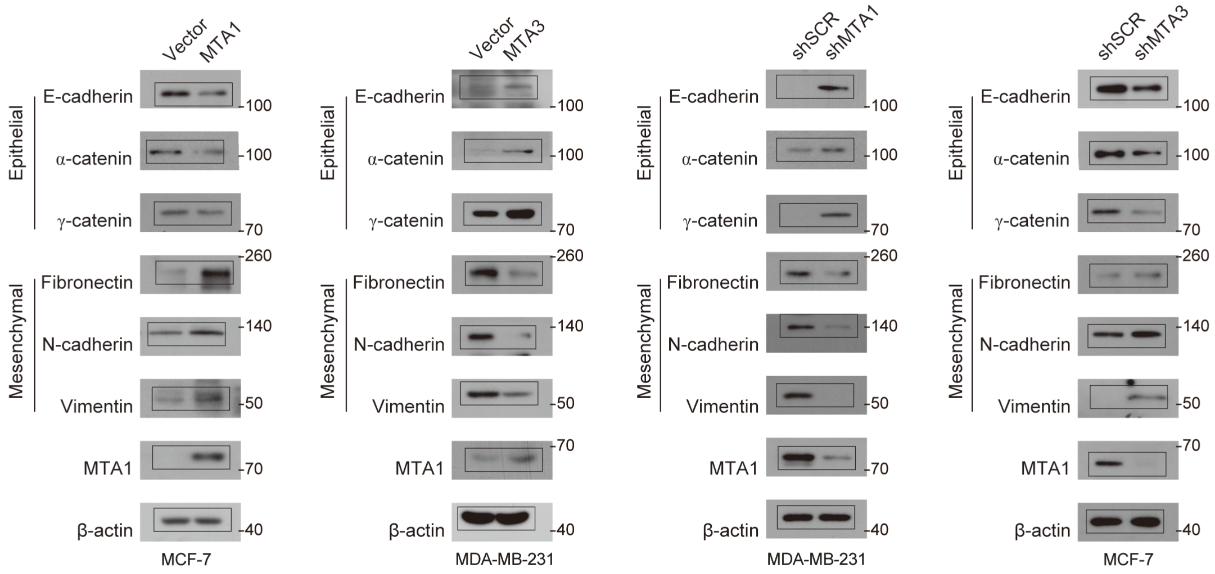

Figure 2E

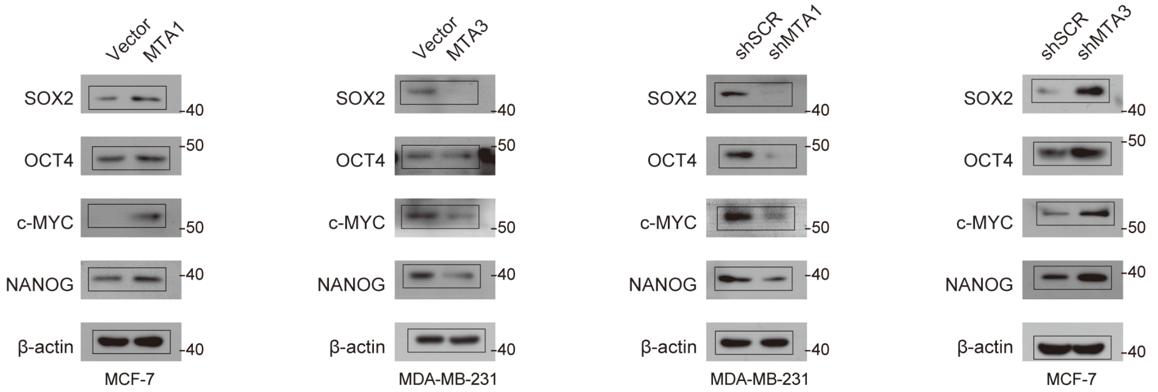

Figure 3E

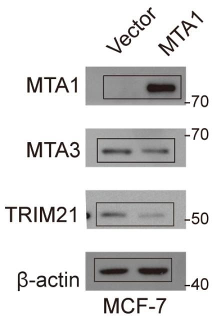

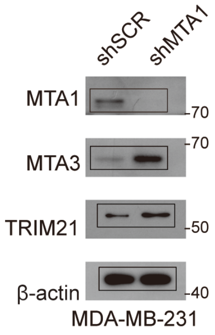

Figure 3G

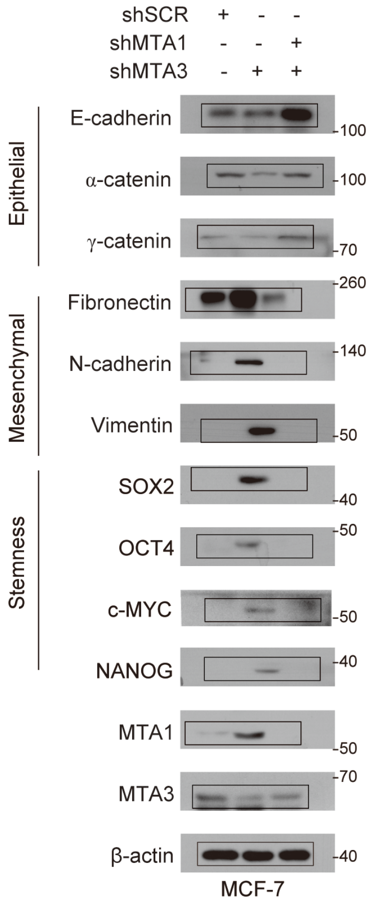

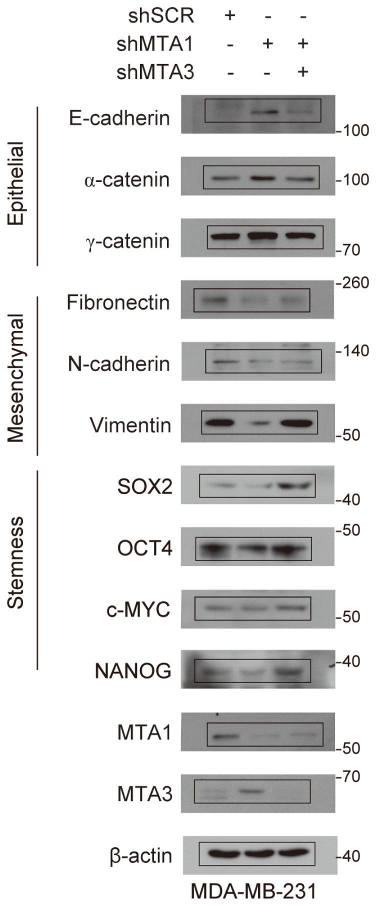

Figure 5D

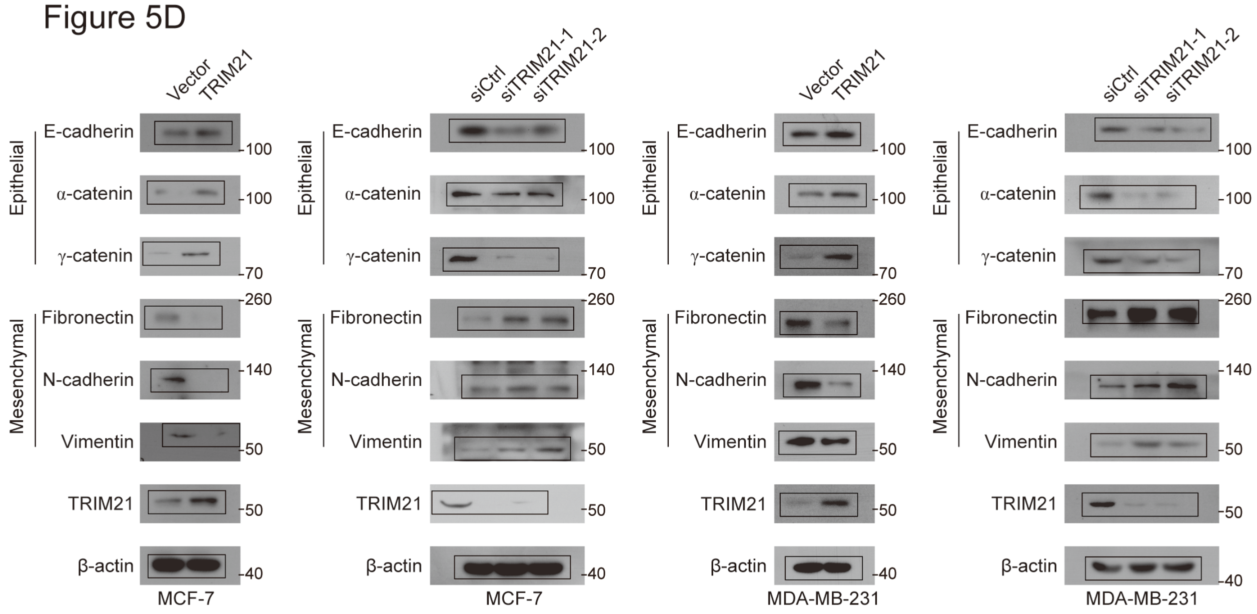

Figure 5F

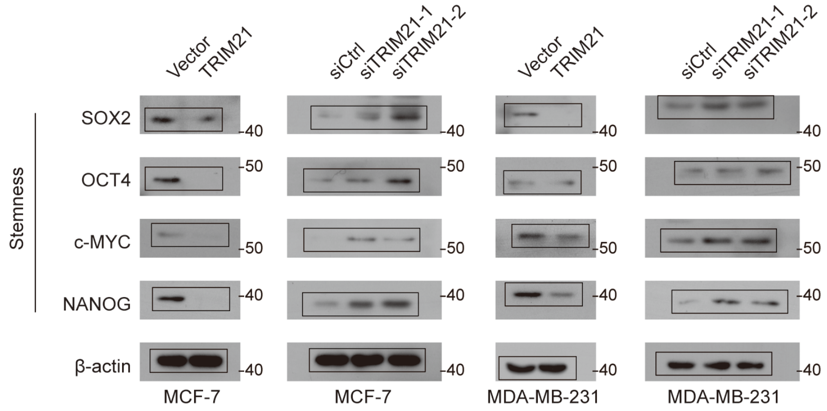

Figure 6A

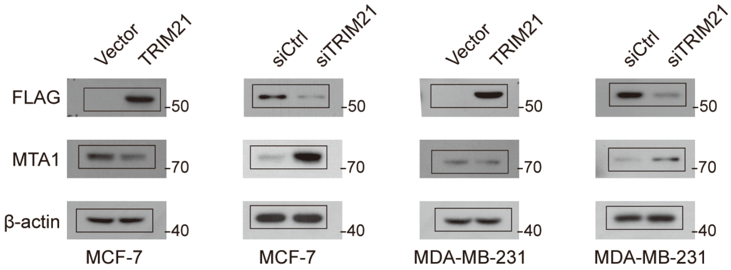

Figure 6B

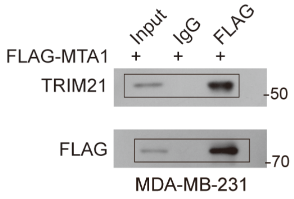

Figure 6C

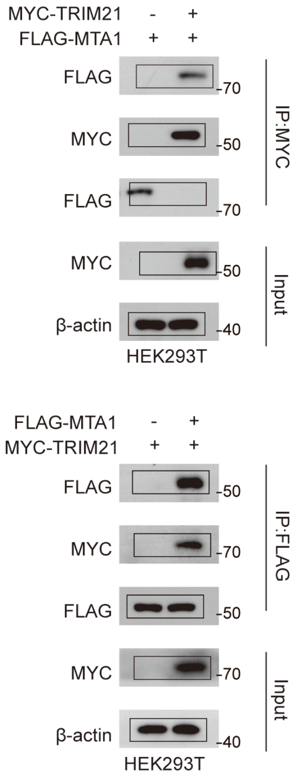

Figure 6D

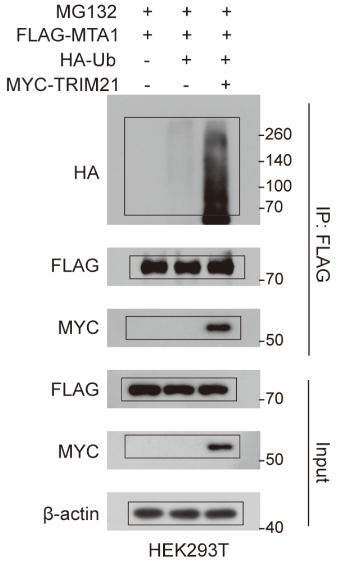

Figure 6E

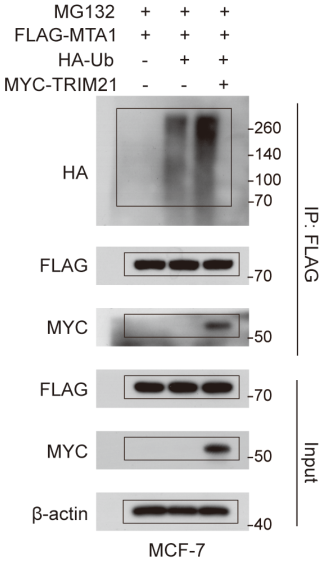

Figure 6F

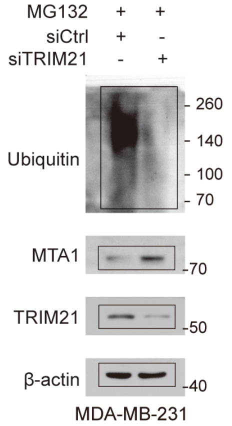

Figure 6G

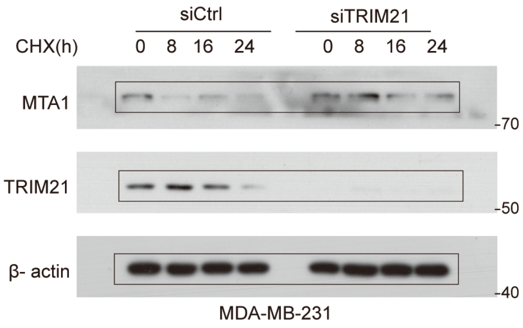

Figure 6H

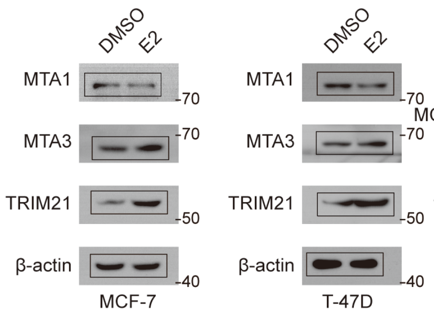

Figure 6I

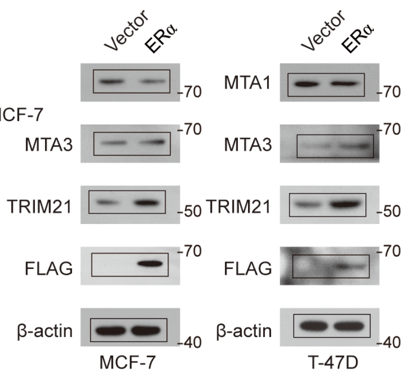

Supplementary Figure 1C

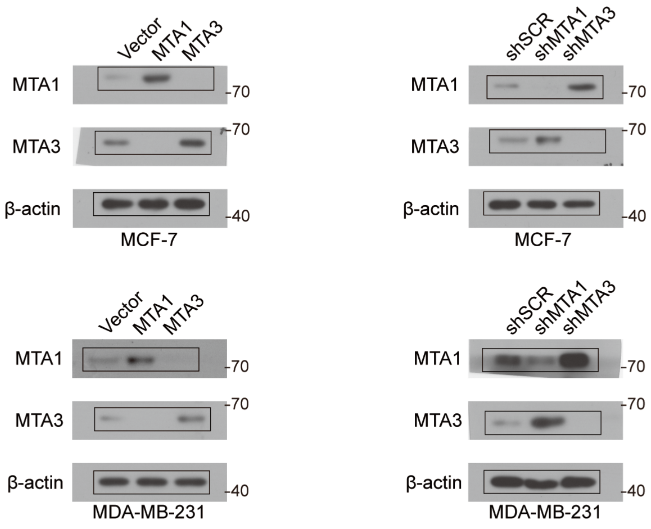

Supplementary Figure 6A

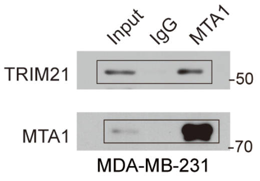

Supplementary Figure 6B

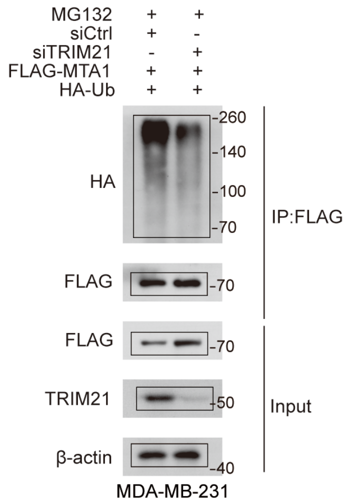

Supplement: Supplementary file 2 — Original western blots [file 41419_2024_6942_MOESM2_ESM.pdf]
